# An octanol hinge opens the door to water transport†

**DOI:** 10.1039/d0sc04782a

**Published:** 2020-12-08

**Authors:** Zhu Liu, Aurora E. Clark

**Affiliations:** Department of Chemistry, Washington State University Pullman Washington 99164 USA zhu.liu@wsu.edu auclark@wsu.edu; Voiland School of Chemical Engineering and Bioengineering, Washington State University Pullman WA 99164 USA; Pacific Northwest National Laboratory Richland Washington 99352 USA

## Abstract

Despite their prevalent use as a surrogate for partitioning of pharmacologically active solutes across lipid membranes, the mechanism of transport across water/octanol phase boundaries has remained unexplored. Using molecular dynamics, graph theoretical, cluster analysis, and Langevin dynamics, we reveal an elegant mechanism for the simplest solute, water. Self-assembled octanol at the interface reversibly binds water and swings like the hinge of a door to bring water into a semi-organized second interfacial layer (a “bilayer island”). This mechanism is distinct from well-known lipid flipping and water transport processes in protein-free membranes, highlighting important limitations in the water/octanol proxy. Interestingly, the collective and reversible behavior is well-described by a double well potential energy function, with the two stable states being the water bound to the hinge on either side of the interface. The function of the hinge for transport, coupled with the underlying double well energy landscape, is akin to a molecular switch or shuttle that functions under equilibrium and is driven by the differential free energies of solvation of H_2_O across the interface. This example successfully operates within the dynamic motion of instantaneous surface fluctuations, a feature that expands upon traditional approaches toward controlled solute transport that act to avoid or circumvent the dynamic nature of the interface.

## Abstract 2

Despite their pharmacological relevance, the mechanism of transport across water/octanol phase boundaries has remained unexplored. Octanol molecular assemblies are demonstrated to reversibly bind water and swing like the hinge of a door.

## Graphical abstract

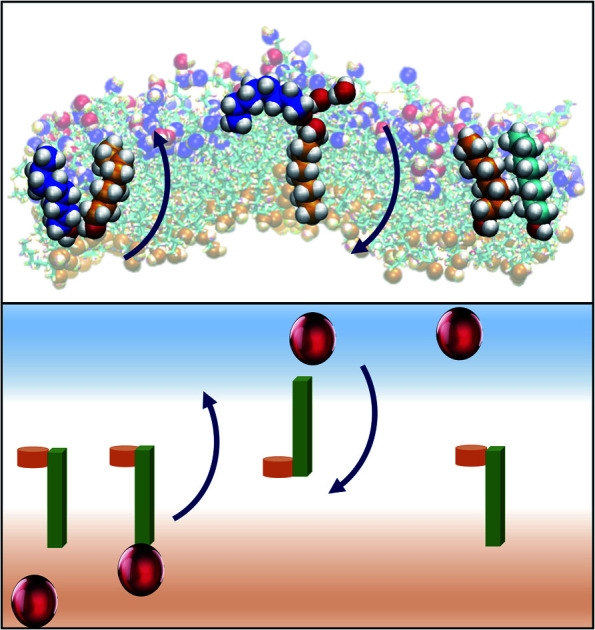

## 1 Introduction

Liquid/liquid interfaces often act as kinetic gatekeepers for solute transport. This can occur in the context of biological systems, where cell membranes control the uptake of pharmacologically active agents, or in separations and purification processes at the industrial scale using methods like solvent extraction. There are few examples of systems that execute a solute transport strategy based upon controlled molecular-scale motion, for instance, lipid bilayer macrostructures (*e.g.* transmembrane proteins) that undergo activation and conformational changes to facilitate solute transport.^1–3^ Alternatively, “catch and release” strategies for separation of complex mixtures have been developed that utilize photoswitching extractant molecules that are selective for specific solutes under different conformational states interconverted by light.^4–8^ Within these cases, the highly dynamic motion of the instantaneous liquid/liquid interface is dampened or avoided in order for the transport mechanism to operate. The lipid bilayer dampens thermal roughness and imparts mechanical stability that supports the function of the embedded macrostructure. The molecular switch extractant utilizes the liquid/liquid interface solely as a depot for the selected solutes.

*A priori*, it may not be necessary to moderate or avoid dynamic surface fluctuations to rationally design a transporting interfacial molecular assembly. In this case, the molecular system would have controlled and reversible movement between two different states that performs the function of solute transport. Within solvent extraction, there are several examples that demonstrate collective motion of the instantaneous surface during solute transport. “Fingers” or “protrusions” are considered amplifications of surface roughness into new structural motifs that facilitate passage of ions across the interface.^9–12^ Modulating protrusion formation has recently begun to be explored; surfactants like tributyl phosphate amplify interfacial heterogeneity and increase the likelihood of water protrusions that are the dominant mechanism for water transport.^13^ Anecdotal evidence from molecular dynamics (MD) implicates protrusions as the transporting mechanism of metal–ligand complexes and larger solutes across oil/water phase boundaries.^14,15^ Although protrusion-based mechanisms may exhibit collective motions, straightforward reaction coordinates have yet to be identified (the closest examples being the recent work of Kikkawa *et al.*^16,17^).

**Fig. 1:**
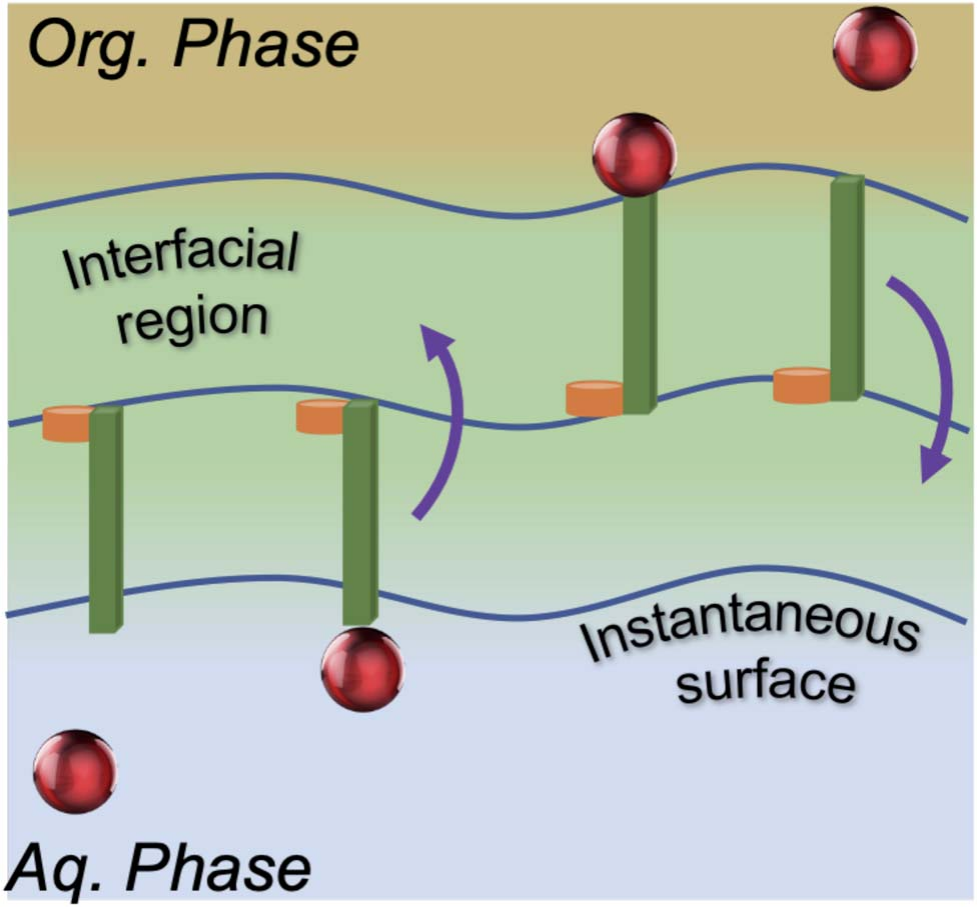
Model of an interfacial hinge for solute transport.

Accordingly, what could be the potential characteristics of a transporting, reversible, molecular assembly that coexists with instantaneous surface fluctuations? One option is a hinge, where one end is embedded within the interfacial region, but the other end has sufficient steric freedom to dynamically traverse the phase boundary to reversibly transport molecules between the two immiscible solutions (Fig. 1). This requires organization of the interface beyond the instantaneous surface, yet at the same time, the interfacial structure should not be too organized otherwise the swinging end of the hinge would be sterically hindered. Metastable configurations are also necessary at both extremes of motion (the organic and aqueous phases) to enable solute binding and release events. If such configurations are accessible under equilibrium conditions, then the differential solubility of the solute across the instantaneous surface acts as a driving force. However external stimuli could also be employed to access the necessary two-state configurations that support the transport mechanism and create a concentration gradient that moves the entire system energetically uphill. Irrespective of this, the combined features of such a system would have a double well potential as a suitable energetic representation, akin to that employed for molecular shuttles and switches.^18^

This work reports the serendipitous observation of a biphasic system that meets the aforementioned requirements under equilibrium conditions, namely, solute transport across the water/octanol interface. This system has significant relevance to the separation and pharmacology communities. Octanol is widely employed as a phase modifier that alters transport kinetics within industrially relevant solvent extraction systems. Additionally, the solute water/octanol partition coefficient is a broadly accepted measure of lipophilicity and octanol is assumed to be a mimetic surrogate for more complicated phospholipids that comprise bilayer membranes.^19–27^ Over 300 peer-reviewed publications have been in print within the last two decades (Google Scholar), with significant recent growth of simulation studies of water/octanol biphasic systems. Although mass transport/kinetic models have been developed for select systems,^28,29^ and non-equilibrium and potential of mean force simulations have been employed to understand energetic favorability in different bulk phases,^30^ the equilibrium transport mechanism has not (to our knowledge) been examined at a molecular level (the scope of studies from more than 100 simulation-based publications is presented in Table S1†). To elucidate the mechanism of H_2_O transport across the liquid/liquid phase boundary, classical MD simulations employed a benchmarked set of interaction potentials that reproduced both the experimental mole fraction water solubility in octanol (0.26 (ref. 31)) and the interfacial tension (7.83 ± 0.80 (ref. 32)), as well as key experimental observations regarding the interfacial structure associated with octanol orientation and partial bilayer formation.

Thorough statistical analyses verify that nearly all H_2_O that migrates from bulk water, through the interface, and into bulk octanol, does so using collectively organized octanol clusters that swing across the interface by reversibly picking up water at the instantaneous surface and depositing them at a semi-bilayer of octanol. From there, water as part of a “bilayer island” may diffuse into bulk octanol or be returned to the instantaneous water surface by the reverse transport process. The water transport mechanism identified here sharply contrasts with that identified by MD studies of protein-free lipid membranes (*via* water defects and the associated formation of water pores^33^) and as such clearly limits octanol as a mechanistic surrogate for solute transport (although other elements of the proxy may hold). The “hinge” mechanism for water transport across the water/octanol phase boundary is further well-reproduced by Langevin dynamics based upon a double well potential energy function. In this case, favorable inter-octanol hydrogen bonding interactions support collective organization of the bilayer islands that have a relatively low barrier toward oscillatory motion, while the high solubility of water in octanol causes stable minima at the instantaneous surface of water with octanol, as well as within the semi-bilayer structure adjacent to, and in dynamic equilibrium with, bulk octanol.

## 2 Methods

### 2.1 Force field benchmarking and simulation protocol

#### Force field benchmarking

Extensive benchmarking of the force field implementation has been performed and is based upon the reproduction of two macroscopic equilibrium properties (the interfacial tension and the mole fraction solubility of water in octanol) as well as experimental resonance enhanced second-harmonic generation studies that have elucidated octanol orientation and organizational features at the interface.^36^ Finite size effects were also examined by monitoring the convergence of all properties to a system box size, where *z* = 70–170 Å and *x* = *y* =40–98 Å (Table S2†).

The four octanol and water force field combinations considered represent the most prevalently employed ones within the literature for bulk and limited interfacial studies: GROMOS54A7/SPC-E,^37^ GAFF/TIP4P-EW,^38^ GAFF/TIP3P,^38^ and OPLS/TIP3P.^39^ The resulting data compared to two prior studies are presented in Table 1. Importantly, there is a strong correlation between the calculated interfacial tension and the associated solubility of water in octanol.^40–42^ The GROMOS54A7/SPC-E set was observed to reproduce the correct solubility and interfacial tension, with excellent agreement being observed for the interfacial structure, as described in Section 3.1, and based upon the associated intermolecular pairwise interactions (Table S3†). Using this set of force field parameters, the GROMACS simulation package^43^ was employed to study the equilibrium properties of the water/octanol interface in a rectangular simulation cell under 3-dimensional periodic boundary conditions (Fig. S1†). The simulation cell contains 2938 octanol and 12 836 water molecules. The nonbonded Lennard-Jones interactions are treated with a spherical cut-off *r*_c_ = 15 Å while the long-range Coulomb interaction is evaluated using Ewald summation^44,45^ with a relative error of 10^−8^. The integration time step is set to 2 fs. The periodic lengths are *L*_*x*_ = *L*_*y*_ = 79.68 Å and *L*_*z*_ = 173.44 Å after equilibration. These dimensions are equilibrated to a pressure *P* = 1 atm and temperature *T* = 300 K *via* the isothermal-isobaric (*NPT*) ensemble for 250 ns, where the Parrinello–Rahman^46^/Nosé–Hoover^47,48^ algorithm with a pressure/temperature coupling time constant of 0.4 ps/2 ps is chosen. Equilibrium is ascertained through monitoring a number of properties, including the interfacial tension (Fig. S2†), concentration of water in the octanol phase (Fig. S3†), and number of transport events between the two phases (*vide infra*). Ergodicity is demonstrated by the equal incidents of forward and reverse transport processes (Tables S5 and S6†). After equilibration, a subsequent 30 ns production simulation is executed *via* a canonical (*NVT*) simulation with a coupling time of 1 ps and configurations recorded every 10 ps. In the case of fast motions (*i.e.* hydrogen bond formation/breakage), additional sampling was performed every 20 fs for 150 ps. All analyses from the production run were performed with block averaging (using three blocks) to quantify statistical significance.^49^

**Table 1:** Summary of intermolecular interactions, mole fraction solubility of water in octanol, and interfacial tension of the liquid/liquid interface predicted with different octanol/water models. CG-MARTINI is the coarse-grained model reported by Ndao *et al.*^34^ Polarized FF and D–C denote the polarizable octanol force field and polarizable Dang–Chang water model reported by Wick and Chang.^35^ The experimental data are from the studies by Šegatin and Klofutar^31^ and Demond and Lindner.^32^

Models (octanol/water)	Intermolecular interactions (kcal mol^−1^)			Mole fraction solubility	Interfacial tension (mN m^−1^)
	Octanol–octanol	Water–water	Octanol–water		
GROMOS54A7/SPC-E	−82 164.65	−135 214.37	−17 139.2	0.26	7.83 ± 0.80
GAFF/TIP4P-Ew	−74 534.51	−138 135.00	−8892.07	0.11	16.28 ± 0.33
GAFF/TIP3P	−85 428.77	−115 686.56	−10 519.85	0.16	11.57 ± 0.50
OPLS/TIP3P	−24 240.02	−75 472.20	−4499.79	0.14	14.35 ± 1.70
CG-MARTINI/CG-MARTINI	—	—	—	0.15	19.7
Polarized FF/D–C	—	—	—	0.26	3 ± 6
Expt.	—	—	—	0.27	8.5

The interfacial tension *γ* is calculated with the Kirkwood and Buff pressure-tensor method^50^1
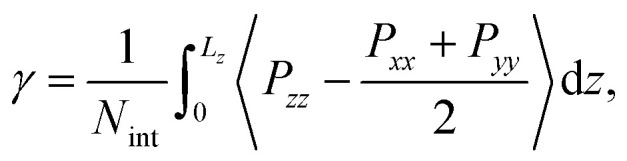
where *P*_*zz*_ is the normal pressure-tensor component, *P*_*xx*_ and *P*_*yy*_ are the tangential components along the respective directions and *N*_int_ is the number of interfaces (2) in the periodic simulation box. The mole fraction solubility of water in the octanol phase is calculated by taking the average densities of water and octanol in the octanol-rich phase.

### 2.2 Analyses

#### The ITIM instantaneous interface

In this work, all hydrogen bond (HB) interactions are analyzed using the ChemNetworks package,^51^ in which H_2_O and octanol molecules are converted to nodes, with any existing hydrogen bond between them as an edge connection. HBs for water–water, water–octanol or octanol–octanol are all defined by an O⋯O distance with a value less than 3.5 Å and an H–O⋯O angle of 0–30°. The strong association of the hydrophilic octanol with water complicates the interpretation of interfacial properties based upon a Gibbs dividing surface (GDS) reference position within the interfacial region. The intrinsic interface (or instantaneous surface) has thus been examined and identified using the Identification of Truly Interfacial Molecules (ITIM) algorithm.^52^ The suggested probe sphere radius of 1.5 Å with a grid spacing of 0.2 Å was employed. As illustrated in Fig. S1,† there exists a non-negligible number of water molecules within the octanol-rich interfacial region. Thus, H_2_O that is distributed in the bulk octanol phase will be mistakenly counted as “truly” interfacial H_2_O in the algorithm. To avoid this issue, a density based cluster approach (Pytim DBSCAN^53^) is adopted to separate the H_2_O molecules that have penetrated the octanol phase *vs.* the truly interfacial H_2_O molecules that remain connected to the aqueous phase *via* a hydrogen bond network. Octanol molecules are counted as truly interfacial if their –OH is hydrogen-bonded to at least one truly interfacial H_2_O molecule.

#### The Willard–Chandler instantaneous interface

The interfacial surface area of the system is determined using a procedure of continuous representation of a discrete instantaneous configuration of surface water molecules proposed by Willard and Chandler.^54,55^ This procedure provides a reliable definition of the relevant spatial fluctuations in space and time of the interface location, which are otherwise averaged out by using the Gibbs dividing surface. We adopt the suggested coarse-graining length of 2.5 Å and 90% water bulk density criterion for obtaining the Willard–Chandler surface of water.^55^ Ensemble average values of the interfacial area are obtained by averaging the individual areas of the instantaneous Willard–Chandler interface.

#### Identification of bilayer islands and organized octanol clusters during water transport

Pytim, a python package that implements cutoff-based clustering and the DBSCAN density-based cluster algorithm, was employed for the cluster search to identify hydrogen bonded clusters consisting solely of octanol or octanol and water and their position within the interfacial region during water transport.^53^ Octanol or water molecules are considered to be in the same cluster if their O-atoms are closer than 3.5 Å, which is the O⋯O distance criterion for a HB.

### 2.3 Langevin dynamics

Many reported molecular machines, switches, and shuttles have simple potential energy landscapes that underpin their fundamental motions.^56^ The ability of a simplified potential energy surface to reproduce the observed water transport from molecular dynamics was tested using a Langevin dynamics (LD) formalism. Therein, for a system of *N* particles with masses *M*, with coordinates *X* = *X*(*t*) that constitute a time-dependent random variable, the equation of motion is 
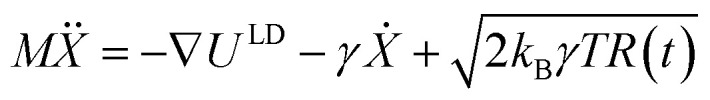
, where *U*(*X*) is the particle interaction potential; ∇ is the gradient operator such that −∇*U*^LD^(*X*) is the force calculated from the particle interaction potentials; the dot is a time derivative such that 
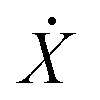
 is the velocity and 
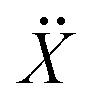
 is the acceleration; *γ* is the friction coefficient; the third term is the noise term with *T* as the temperature, *k*_B_ as Boltzmann's constant, and *R*(*t*) as a delta-correlated stationary Gaussian process with zero-mean.

**Fig. 2:**
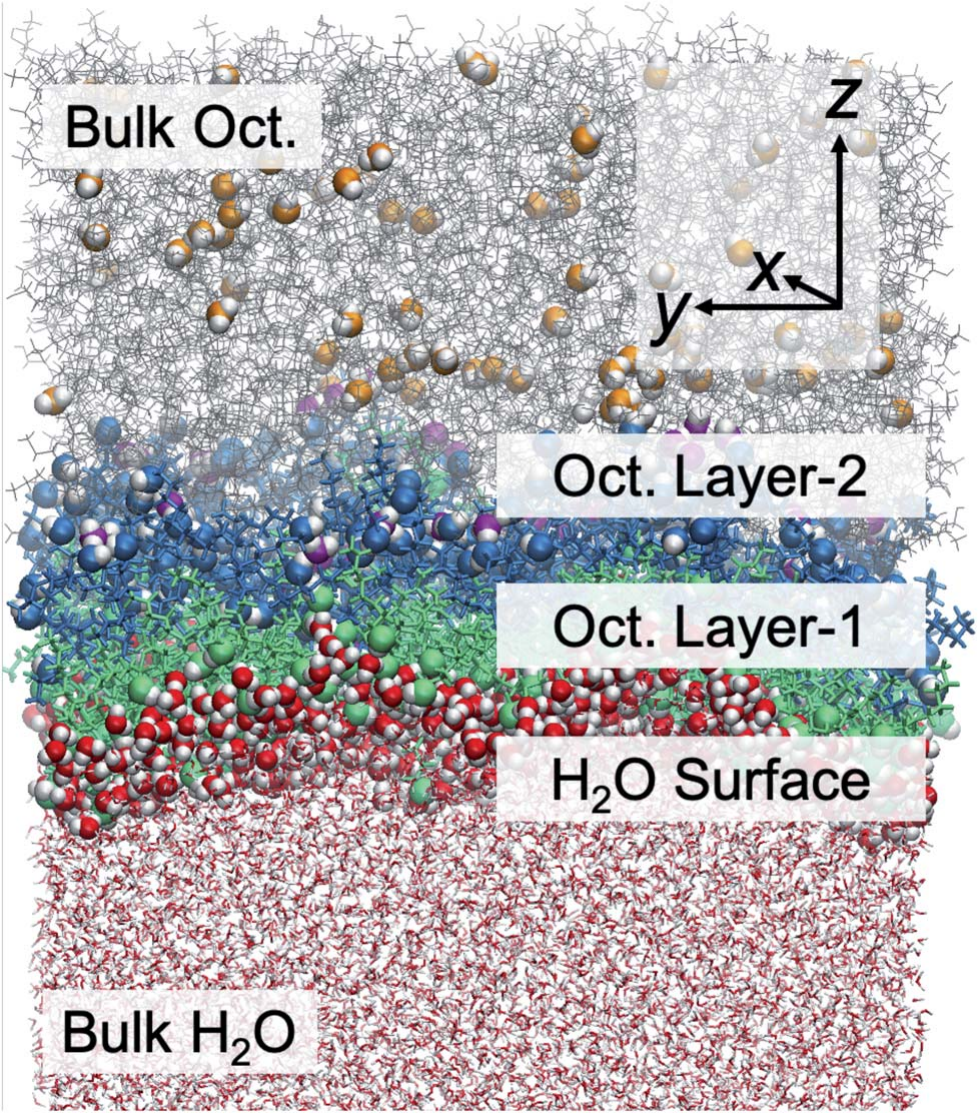
Schematic representation of the water or octanol molecules in different regions, namely bulk water, Layer-1, Layer-2, and bulk octanol phases. Bulk water molecules are depicted with red lines. Water molecules in Layer-1, Layer-2, and the bulk octanol phase are highlighted with red, purple, and orange oxygens, respectively. Octanol molecules in Layer-1 are highlighted with green bonds for alkyl carbon tails and green spheres for oxygens in the hydroxyl head group. The alkyl tails are drawn in blue with the hydroxyl oxygen highlighted as blue spheres for octanol molecules in Layer-2. Bulk octanol molecules are depicted with gray lines.

Several different fitted double well potential energy functions were employed, as described in the ESI,† based upon the differing Arrhenius parameters for determining the respective activation barriers, *E*_a_, for water transport (*vide infra*). The potential energy landscape for the LD simulation is created under the following requirements. First, the activation energy barrier of the potential landscape is located at *x* = *z* = 0. Second, the left well is located at *z* ∼ −0.868 nm (*x* = 0), whose value is taken from the average molecular length of octanol in Layer-1 (see Fig. 2 in Section 3.1). At the same time, the average Layer-2 octanol molecular length is set to be the *z* location of the right well, that is *z* ∼ 0.854 nm (*x* = 0). In this way, the double well structure is representative of the head group clusters of octanol and water being transferred between Layer-1 and Layer-2 (Section 3.2). The left ramp potential barrier is adopted from the transport activation energy barrier of water from the octanol Layer-1 region to the Layer-2 region (Section 3.3), while the depth of the right well represents the barrier for transport from Layer-2 to Layer-1. The respective barrier heights were obtained from different Arrhenius pre-factor *A* values and also incorporating the statistical uncertainties from the data obtained from the MD simulation using block averaging. The particle within the Langevin dynamics will experience the double well potential energy, friction and noise forces and evolve its positions by the Langevin integrator. The temperature (300 K) and sample time (1 ps) of the Langevin dynamics simulation are set to the same as the course of the MD simulation. Each Langevin dynamics simulation is run for 100 000 steps to obtain meaningful statistics. The uncertainties are calculated by running the simulation 1000 times and calculating the standard error of the mean.

## 3 Results and discussion

### 3.1 Octanol interfacial structure in the *z*-direction

A molecular scale understanding of the liquid/liquid interfacial structure is essential toward defining and understanding the water transport mechanism. We focus first upon understanding the organization across the interface, along the *z*-direction (Fig. 2). The instantaneous interface has significant distribution in *z* due to local thermal corrugation that exists in combination with longer-range capillary waves. Typical analyses of interfacial organization often begin with density profiles of the respective solvents along *z*, complemented by radial distribution functions of essential functional groups. These data (Fig. S4†), shown and discussed in detail within the ESI,† are the first intimation of density oscillations in the hydroxyl O-atom and methyl carbons that coincide with a bilayer-like structure where the carbon tails of octanol molecules at the instantaneous surface are organized end-to-end with the alkyl tails of octanol in a second layer from the surface. The distance distribution from the hydroxyl O-atom to the terminal C-atom indicates a high probability of the elongated linear configuration (Fig. S5†), consistent across several force fields and prior work.^35^ The octanol bilayer-like structure is depicted in both Fig. 2 and S1.† The orientation profiles of water and octanol molecules as a function of *z* are presented in Fig. S4,† demonstrating that octanol molecules at the interface orient their hydroxyl head groups pointing toward the water phase with a cosine *θ* value around 0.7 (45°). Here *θ* is the angle between the octanol molecular end-to-end vector passing through the hydroxyl H- and terminal C-atoms and the surface normal along the *z* axis. The interfacial orientation is in good agreement with the experimental value reported by Cramb *et al.*,^57^ obtained from second harmonic generation spectroscopy and measured to be 39 ± 10° to the surface normal. Further into the octanol phase, there is a preference for octanol molecules to point their hydroxyl oxygens in the opposite direction with respect to the interfacial octanol orientation, toward the octanol phase. The orientational data support the organization of an octanol bilayer-like region in the vicinity of the liquid–liquid interface, as observed in MD simulations using polarizable forcefields.^35,41^ Based upon these data, we then delineate five regions of the chemical system: the bulk aqueous phase, the instantaneous surface of H_2_O in direct contact with octanol, the octanol molecules at the instantaneous interface with water that have their –OH groups facing the aqueous phase (labelled Layer-1), octanol molecules that form a second semi-organized layer and have their –OH groups facing the organic phase (labelled Layer-2), and the bulk octanol phase. These regions are presented in Fig. 2.

### 3.2 Octanol structure parallel to the interface

**Fig. 3:**
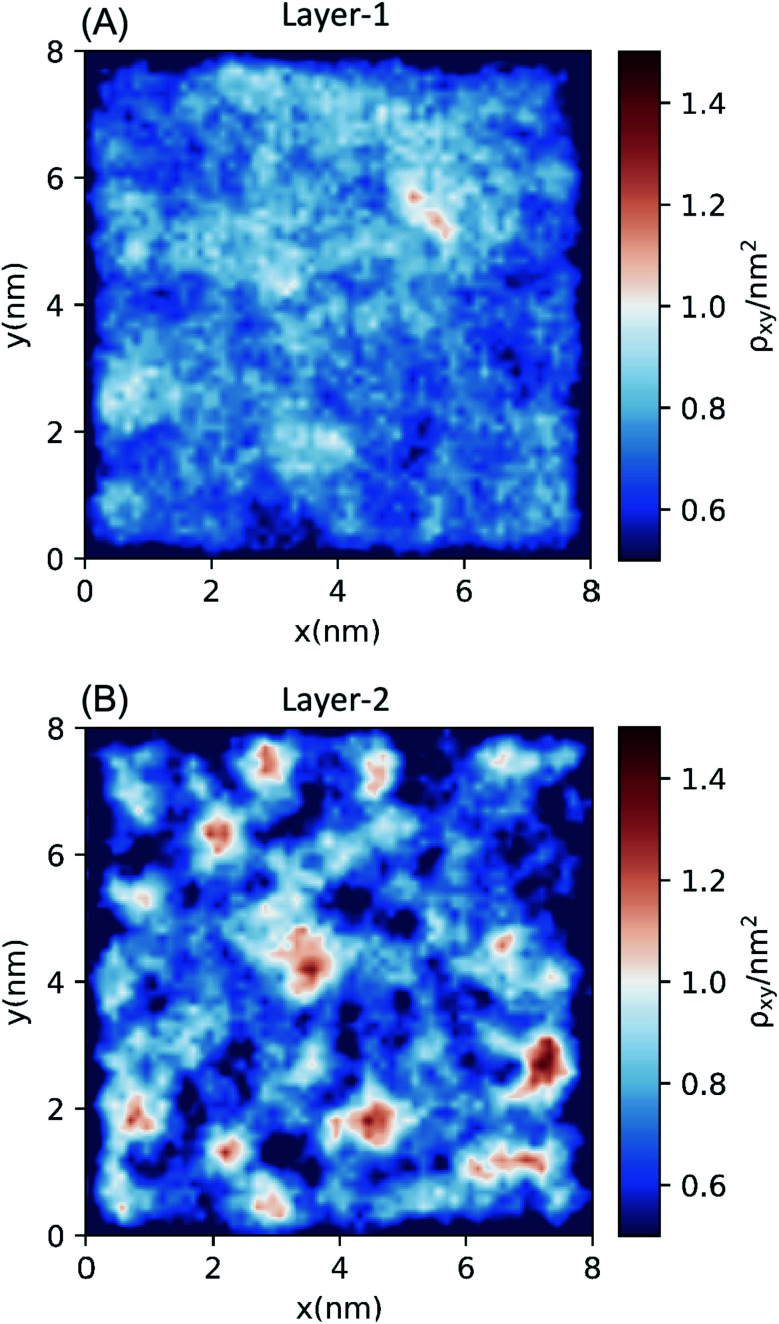
Two-dimensional density distributions of hydroxyl O-atoms of octanol molecules in (A) Layer-1 (the instantaneous octanol surface) and (B) Layer-2 (the second layer from the instantaneous surface).

The bilayer-like structure of the octanol interface can be further quantified by metrics that assess the spatial heterogeneity along (as opposed to across) the surface. Distinct differences are observed between Layer-1 and Layer-2. Within the instantaneous octanol surface (Layer-1), examination of the hydroxyl O-atom density in the *xy* plane (Fig. 3) reveals a fairly homogeneous distribution of octanol with an average packing density of 2.80 oct per nm^2^ (using the Willard–Chandler surface), in reasonable agreement with the experimental value (3.56 oct per nm^2^).^58^ The spatial correlation between octanol molecules in Layer-1 was measured with 2-dimensional radial distribution functions (2D-RDF, Fig. S6†), which exhibits a correlation peak around 2.4 Å, corresponding to the octanol–octanol dimer (held together by a single hydrogen bond), and a second correlation peak at ∼ 4.2 Å that corresponds to the water-bridged octanol–water–octanol dimer, observed in previous studies.^59,60^

In contrast to Layer-1, the *xy* plane density heat map of hydroxyl O-atoms in Layer-2 (Fig. 3B) exhibits significant heterogeneity, with distinct clustering of octanol surrounded by low-density octanol regions. The Layer-2 RDF presents an outstandingly higher peak at ∼2.4 Å than the Layer-1 RDF, and a relatively lower height second peak at ∼4.2 Å. Interestingly, in the Layer-2 2D-RDF, anti-correlation is observed between 5 and 13 Å, which also indicates octanol deficient regions in the second layer of the octanol bilayer. This feature was further analyzed by calculating the excess coordination number (ECN):2
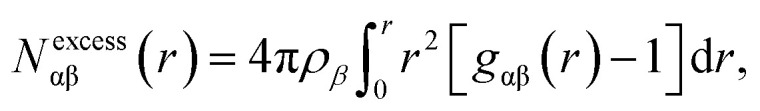
where *g*_αβ_(*r*) is the Kirkwood–Buff *g* factor between species α and β of interest and *ρ*_β_ is the number density of the β component. From a physical point of view, the ECN *N*^excess^_αβ_(*r*) is the difference in the number of β species in the vicinity of a central α species compared to that found in an equivalent volume of the bulk solution. Therefore, a positive ECN value indicates an excess of β species in the vicinity of species α over the bulk distribution, while a negative value will correspond to a depletion of species β surrounding α. In this work, *g*_αβ_(*r*) is just the 2D-RDF *g*(*r*)_oct–oct_ with α, β referring to octanol only. For the coordination of octanol molecules in Layer-1, beyond 5 Å the ECNs around *z* = 0 indicate a uniform octanol distribution at the instantaneous surface. In Layer-2, the relatively large ECN value around 2.4 Å indicates anomalous, higher densities of octanol relative to those in Layer-1. This is consistent with the 2D-RDF, where enhanced peak intensity is consistent with clustering. In the distance range of 5 to 15 Å, negative ECN values correspond to a depletion of the octanol hydroxyl head groups. This indicates significant “gaps” in octanol density between octanol clusters, and thus we adopt the terminology “bilayer islands” for this phenomenon (Fig. 3 and S6†).

Importantly, the bilayer islands cause an enhanced nonpolar alkane-like region in the vicinity of the interface. As described by Steel and Walker^36^ solvatochromic surfactants have been used to probe the change in polarity across the water/octanol interface using resonance-enhanced second-harmonic generation. Their data provided evidence that the water/octanol interface has a region that is less polar than either of the individual solvents, implying a hydrophobic barrier (exposed alkyl groups) between the two bulk polar phases.^36^ At the same time, the data also indicated a membrane-like structure. Prior work using polarizable octanol and water has also predicted a semi-organized second layer of octanol relative to the instantaneous surface,^35^ while most non-polarizable force fields predict a monolayer under equilibrium conditions and mixing/demixing studies sometimes predict a semi-bilayer structure that is dependent upon system configuration.^40,61^ We compared several classical force field representations (Tables 1 and S3†) and analyzed the associated intermolecular pairwise interactions which indicated that strong interactions of the water and octanol hydroxyl group have a dual effect of causing the correct water solubility and stabilizing water rich octanol islands in the semi-bilayer.

### 3.3 Water transport mechanism

**Fig. 4:**
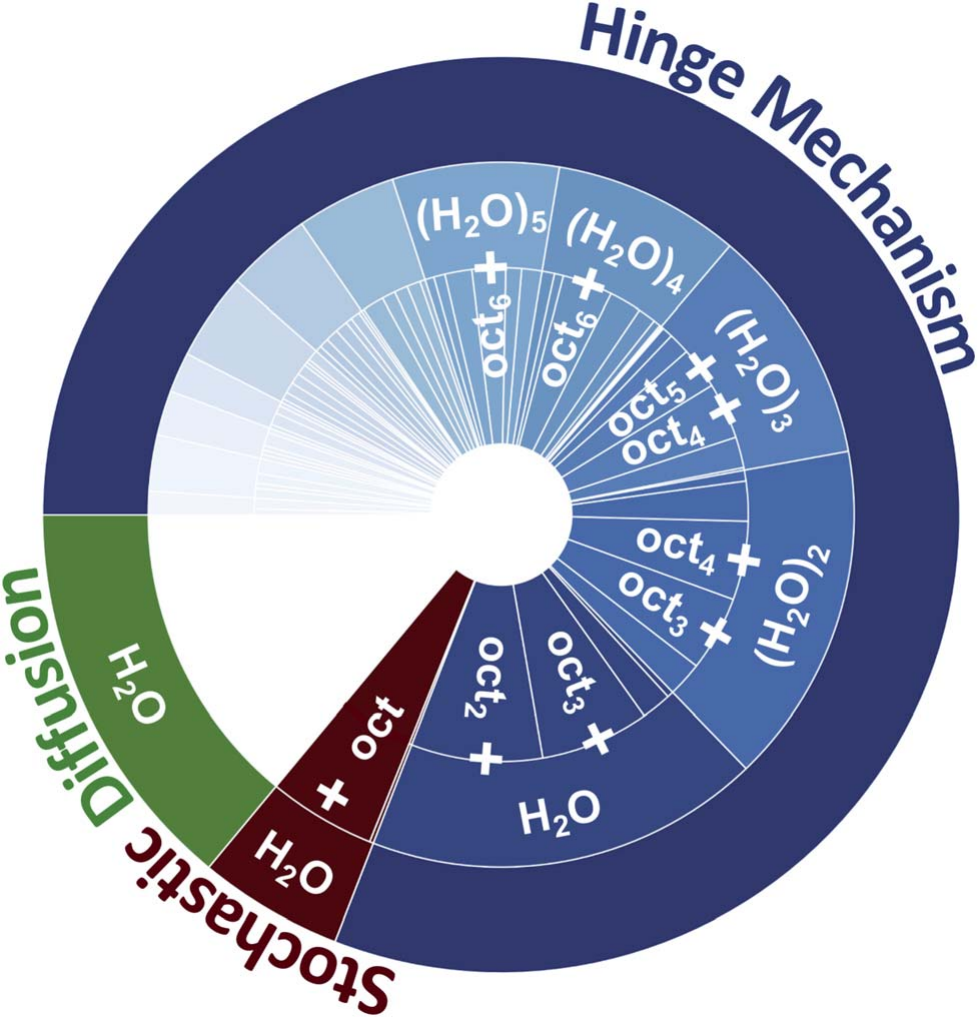
The relative contributions of different water Layer-1 → Layer-2 transport mechanisms (diffusion, stochastic octanol flipping, and the hinge mechanism) observed within the water/octanol system, and the composition of transporting species within each transport mechanism. Water diffusion comprises 14% of all transported water (in green), while 6% is transported by a single hydrogen bond to a stochastically flipping single octanol (in red), and 80% are transported by an octanol molecular hinge that is composed of (oct)_*n*_(H_2_O)_*m*_ clusters (in blue). The relative contributions of each cluster composition are provided within the inner rings of the plot. Only transport species with a percentage above 5% are listed. The Layer-2 → Layer-1 transport is depicted in Fig. S8.†

Initial analysis of the dynamic features of the semi-bilayer structure of the octanol surface reveals characteristics that are reminiscent of analogous protein-free lipid membranes^62^ that have a more significant organizational structure. For example, individual octanol molecules undergo stochastic flipping events between layers (Layer-1 ⇔ Layer-2) as well as diffusive motion to and from Layer-2 and bulk octanol. Within 30 ns of simulation, using 10 ps sampling, 26 650 flipping events were observed, evenly distributed between Layer-1 → Layer-2 and the reverse process (Table S5† and Fig. 4). Interestingly, prior work has demonstrated that water defects and the associated formation of water pores is essential to lipid flipping in protein-free membranes;^33^ however a distinguishing characteristic of octanol flipping is that the stochastic process consists of single octanol molecules that are not hydrogen bonded to H_2_O. Out of the 26 650 flipping events from the 10 ps sampling only 350 are observed to involve an octanol hydrogen bonded to one H_2_O (Table S5† and Fig. 4). The facile nature of the unimolecular flipping process prompted a more detailed investigation using 20 fs sampling, wherein a rate of 1308.70 ± 52.39 events for Layer-1 → Layer-2 and 1309.18 ± 53.52 events for Layer-2 → Layer-1 are observed per 10 ps. To estimate the barrier for flipping, we employed the Arrhenius equation 
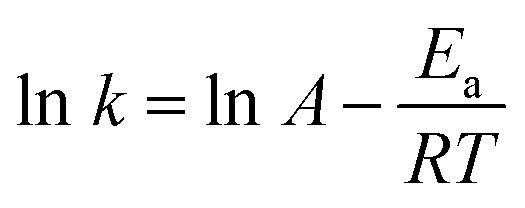
. Here, *k* is the flipping rate constant (using the concentration of octanol in each layer) and *A* is the pre-exponential factor. For *A*, we presume that in order for an octanol to stochastically flip, it must not be hydrogen bonded to any other species, and thus *A* is estimated to be the rate at which octanol within a layer loses its hydrogen bonds to other octanol and H_2_O. Using this model the activation energy barrier is predicted to be 0.21–0.25 kcal mol^−1^ with a ±0.01 kcal mol^−1^ uncertainty (Table S7†). The facile nature of octanol stochastic flipping is not entirely surprising, as prior work has shown that the barrier for lipid flipping can be significantly modulated by alkyl chain length, membrane packing, and the nature of the hydrophilic head group.^63^

Individual water transfer events consist of H_2_O adsorbing to the instantaneous surface from the bulk. This is a diffusion limited process, that has an average rate of 19 161.46 ± 38.32 water molecules per 10 ps (using 20 fs sampling). After adsorption, H_2_O moves from Layer-1 of octanol to Layer-2 of octanol (*via* a mechanism described below) and then diffuses from Layer-2 to bulk octanol. The final step is also rapid, with a rate of 1031.01 ± 30.16 waters per 10 ps. The analogous transfer of H_2_O from octanol to the aqueous phase also occurs as part of the H_2_O partitioning at equilibrium. This work focuses upon the rate-limiting process for water transport between the aqueous and octanol phases, namely Layer-1 ⇔ Layer-2.

Using a combination of hydrogen bond and cluster analyses all events where H_2_O was transported between Layer-1 ⇔ Layer-2 and between Layer-2 ⇔ bulk octanol were analyzed. The equilibrium transport statistics between Layer-1 ⇔ Layer-2 and between Layer-2 ⇔ bulk octanol are collected in Tables S5 and S6† and shown graphically in Fig. 4. Within the 30 ns of equilibrium MD simulation, 477 instances of water diffusion events are observed, where no H_2_O⋯oct hydrogen bonding interactions occur during H_2_O migration across the interface. This comprises 14% of all instances of H_2_O being transferred from the instantaneous surface of water to Layer-2 of octanol. In comparison, 1391 instances of Layer-1 → Layer-2 water transfer events are observed that involve a direct HB between an octanol and H_2_O, which transport a total of 2931 water molecules to Layer-2. Similarly, there exist 1404 transfer events for Layer-1 ← Layer-2 that transport 2850 water molecules back to Layer-1. Importantly, 87.5% of these instances involve the transport of one or more H_2_O molecules to and from a bilayer island by an assembly of octanol molecules, with the remaining 12.5% being the rare instance of one H_2_O molecule hydrogen bonded to a stochastically flipping single octanol molecule. Equivalently, the events that employ octanol assemblies transport 94% of all H_2_O molecules that migrate between Layer-1 ⇔ Layer-2, while only 6% of waters that are transported involve the stochastically flipping octanol. This important characteristic portends a very different mechanism of water transport, one that relies upon collective motion of octanol and water and small networks of intact HB networks to facilitate H_2_O transport across the interface.

**Fig. 5:**
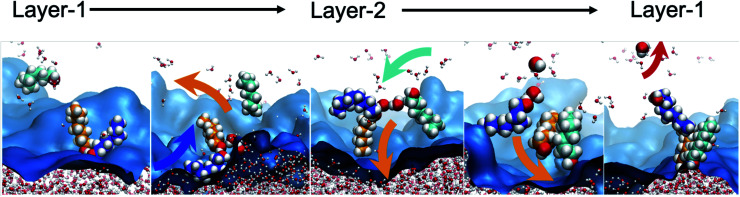
A water transport event *via* the octanol hinge from the aqueous to organic phases across 1.6 ns of trajectory. First H_2_O binds to two octanols, followed by hinge rotation of engaged octanol molecules to Layer-2, and then H_2_O deposition into a bilayer island is observed followed by hinge assembly reorientation (now devoid of water) to Layer-1. Octanol molecules not involved in the event are omitted for clarity. The background blue surface is the instantaneous representation of the water/octanol interface.

To further characterize the distinctive nature of the H_2_O transport by octanol assembly, we first analyze the structural and geometric changes during the transport process, which intimately relates to the bilayer island structural features of the interface. Bilayer islands predominantly consist of 4–5 octanol and 1–3 H_2_O molecules (Table S4†). They are characterized by enhanced water content, as analyzed by their networks of hydrogen bonds between octanol⋯octanol, water⋯water and water⋯octanol (Fig. S9 and S7†). When a water is transferred from Layer-1 → Layer-2, two processes are observed. First, within the dynamically evolving hydrogen bond network of oct⋯oct and oct⋯H_2_O interactions, small octanol–water clusters form that swing from Layer-1 to Layer-2, like the hinge of a door. The transported waters are deposited into predominantly pre-existing bilayer islands whose water concentration scales linearly with the bilayer island size (Fig. S9†). Additionally, entire bilayer islands (or a sub-set of bilayer island octanol molecules) that are devoid of H_2_O are observed to have the hinge-swinging motion from Layer-2 to Layer-1, whereupon one or more H_2_O molecules bind to the self-assembled cluster. Then, the cluster swings back from Layer-1 to Layer-2 to deposit the water in this region. In fact, these two observations are likely the same process, as once a self-assembled octanol cluster is present in Layer-1 it becomes indistinguishable (using our analyses) from non-transporting hydrogen bonded octanol groups, until water binding and transport occur. The reverse process, meaning H_2_O binding to a bilayer island and water deposition into Layer-1 is observed with nearly equal statistics. A portion of the equilibrium oscillation between layers is illustrated in Fig. 5, as: (1) a water binding event to form the transporting assembly (oct)^L1^_*n*_ + H_2_O^L1^_*m*_ → (oct)_*n*_(H_2_O)^L1^_*m*_, (2) hinge swinging transport between layers (oct)_*n*_(H_2_O)^L1^_*m*_ → (oct)_*n*_(H_2_O)^L2^_*m*_, (3) water release (oct)_*n*_(H_2_O)^L2^_*m*_ → (oct)^L2^_*n*_ + H_2_O^L2^_*m*_, and (4) octanol assembly devoid of water swinging back to the original layer (oct)^L2^_*n*_ → (oct)^L1^_*n*_. The analogous change to the octanol dipole orientational angle is depicted in Fig. S10.† This mechanism, hereafter referred to as the “hinge mechanism”, is validated through a number of analyses.

On an average 3.71 ± 0.05 octanol molecules are involved in the Layer-1 → Layer-2 transfer of 2.28 ± 0.11 H_2_O molecules per transfer event, while 3.65 ± 0.05 octanol molecules participate in Layer-1 ← Layer-2 transfer of 2.24 ± 0.07 H_2_O molecules per transfer event. These are within the statistical error and show that the initial and final “states” of the octanol cluster-based hinge are approximately the same (ignoring any effects due to variations in solvation on either side of the phase boundary). The distribution of hinge transporting assemblies can be seen in Fig. 4, which labels the three ways in which water can be transported across the water/octanol phase boundary (diffusion 14%, stochastic flipping 6%, and the hinge mechanism 80%, respectively). All percentages are calculated basing on the statistics in Table S5.†

In addition to the fundamental variance in the composition of the transporting species, and the fact that water transport *via* the hinge mechanism is correlated with the presence of bilayer islands, the energetics of water transport *via* the hinge mechanism is decidedly different from stochastic octanol flipping processes. The relatively loose packing of octanol in Layer-1, combined with its highly dynamic nature, where stochastic octanol flipping creates vacancies in Layer-1 organization, presumably facilitates accommodation of the hinge molecular assemblies and leads to reasonable barriers for transport. Although each transporting hinge is a relatively stable (oct)_(2−*n*)_ species, it is useful to consider the simplified stoichiometry of the transporting reaction on a per H_2_O molecule basis so as to estimate the transport barrier. Dividing the average composition of the transferring octanol island by the average number of H_2_O molecules transferred yields Layer-1 (oct)_1.62±0.06_ + H_2_O ⇔ (oct)_1.63±0.03_ + H_2_O in Layer-2. To be able to employ the Arrhenius equation using this chemical reaction is, however, not possible, as to determine the rate constant, both the rate of transfer and the total concentration of all reactants and products are needed. Within the analyses performed only the successful transfer processes are able to be identified, but not unsuccessfully transferring hinge molecular assemblies. Thus, ascertaining the total concentration of all hinge molecular assemblies represents a significant challenge. Instead, we employ the Arrhenius equation for the following reaction:H_2_O^L1^ + 1.63(oct)^L1^ ⇔ H_2_O^L2^ + 1.63(oct)^L2^This reaction encompasses both the formation of the (oct)_1.63_(H_2_O) reactive hinge assemblies from all water and octanols in a layer and the transport of water in the hinge form, where the concentrations of all reactant and product species are known. Further, reasonable estimates of the prefactor *A* can be made for both the forward and reverse transport processes using (1) the rate of new hydrogen bond formation between water–octanol (in that a water–octanol cluster cannot form without the formation of a HB) or (2) the rate of new waters that adsorb to the surface and hydrogen bond with octanol. With these data, a small range of similar but statistically different *E*_a_ values are obtained for the Layer-1 → Layer-2 *versus* the Layer-2 → Layer-1 transport process (Table S8†). The largest variation between the two activation barriers is *E*^L1→L2^_a_ = 6.18 kcal mol^−1^ and *E*^L2→L1^_a_ = 4.22 kcal mol^−1^, whereas the smallest variation is *E*^L1→L2^_a_ = 6.00 kcal mol^−1^ and *E*^L2→L1^_a_ = 5.60 kcal mol^−1^.

**Fig. 6:**
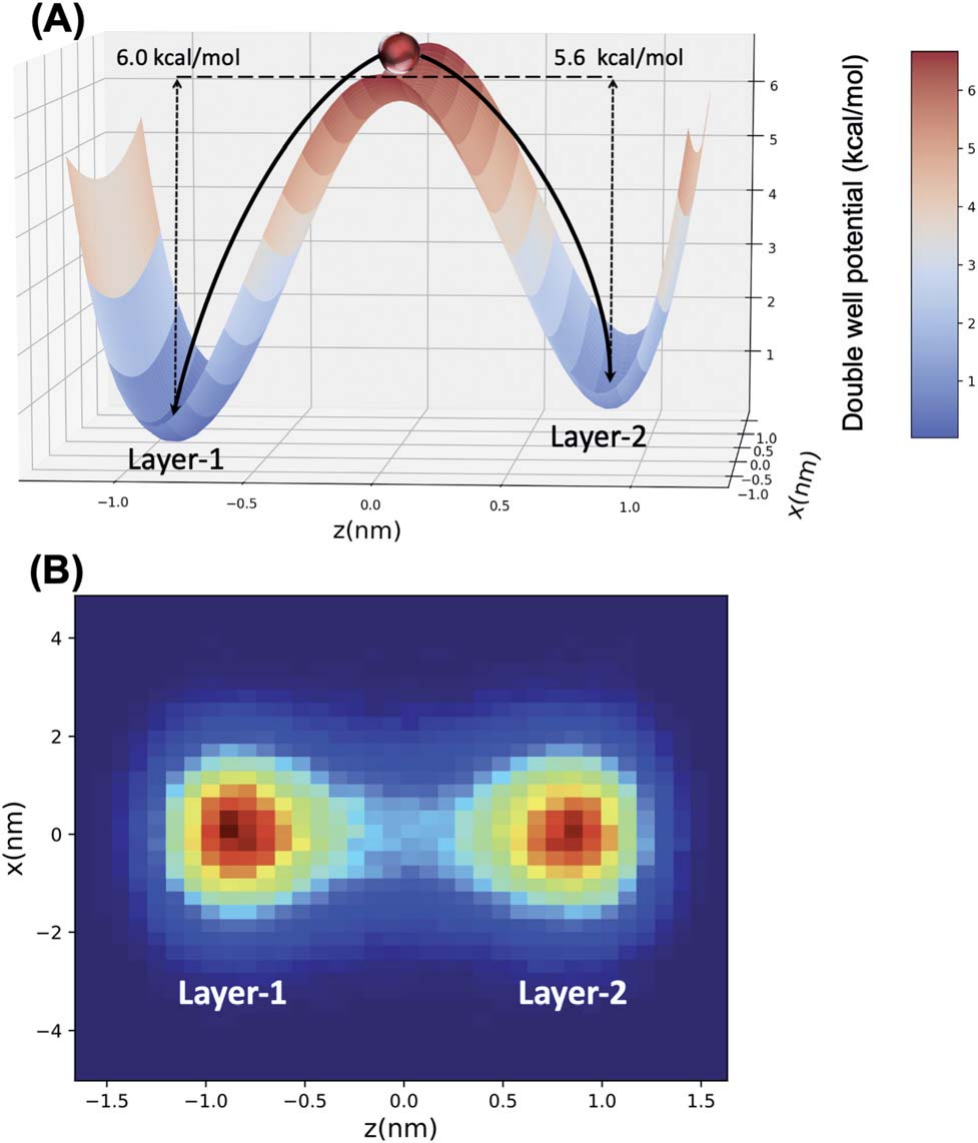
(A) The double potential well aligned along the *z*-direction has a barrier separating two minima located in Layer-1 and Layer-2 regions of the water/octanol interface. *E*_a_ values obtained from Arrhenius fitting to the observed water transport events in the MD trajectory. (B) A particle traverses under the double-well energy landscape *via* Langevin dynamics simulation, where red and blue regions correspond to high and low probabilities of particle concentration (due to transport between the two minima).

Langevin dynamics simulations were then performed using a double-well potential and the estimated *E*_a_ values, where the ratio of transferred particles (H_2_O) between each minimum (representing Layer-1 and Layer-2, respectively) was examined to test the fidelity of the double-well model to mimic water transport. Using block averaging, the ratio of H_2_O transferred from Layer-1 → Layer-2 over the reverse process is 1.03 ± 0.02 from the molecular dynamics simulations. In the case of the LD simulation, the best agreement with MD data is obtained with an *E*^L1→L2^_a_ = 6.00 kcal mol^−1^ and *E*^L2→L1^_a_ = 5.60 kcal mol^−1^, wherein a transport ratio of 1.16 ± 0.11 is obtained. The double-well potential, alongside the LD trajectory concentrations in each minimum, are depicted pictorially in Fig. 6. These data clearly demonstrate that the hinge transport mechanism is well-modeled using a simplified double-well potential energy landscape, with the forward and reverse mechanism being essentially identical.

## 4 Conclusions

The water/octanol interface is of fundamental importance to separations science, both in the context of biological partitioning of solutes across cell membranes as well as industrial purification. To date the mechanism for solute transport has not been elucidated. The current MD study reveals collectively organized transporting units of octanol at the interface and a well-defined and reversible mechanism that is reproduced using Langevin dynamics employing a double well potential. The ability of the transporting octanol “hinge” motif to successfully operate within the surface fluctuations of the interface is a testament to the balance of hierarchical organization and energetic driving forces associated with the mutual solubility of water in the biphasic system. Although the instantaneous surface of octanol in direct contact with water is highly organized, a second bilayer-like organization exists a layer away from the interface that consists of hydrogen bonded octanol islands. The dynamic fluctuations within the interfacial region provide sufficient steric flexibility for bilayer islands (or sub-components) to swing to and from the instantaneous surface to the second layer of octanol in equilibrium with the bulk octanol phase. The reported transport mechanism is further distinct from recent reports regarding water transport through protein-free lipid membranes. The current work thus provides additional context to the generally accepted paradigm that the water/octanol system is a surrogate of biological membranes as it pertains to lipophilicity, solute partitioning, and kinetics of partitioning. Ongoing work is investigating how the well depths, widths, and concurrent barrier height in the double well model of the hinge mechanism can be modulated by parameters of the interfacial structure and intermolecular forces. These include surfactant packing within the instantaneous surface, the second layer of the surface, and the strength of surfactant⋯surfactant and surfactant⋯solute interactions.

## Conflicts of interest

There are no conflicts to declare.

## Supplementary Material

SC-012-D0SC04782A-s001
