# Supplementary material for: An octanol hinge opens the door to water transport
Source: Chem Sci. 2020 Dec 8;12(6):2294–303. doi: 10.1039/d0sc04782a (PMC8179308; doi:10.1039/d0sc04782a)
Supplement: SC-012-D0SC04782A-s001 [file SC-012-D0SC04782A-s001.pdf]

Electronic Supplementary Material (ESI) for Chemical Science.
This journal is © The Royal Society of Chemistry 2020

Supporting Information for
An Octanol Hinge Opens the Door to Water Transport

Zhu Liu* and Aurora E. Clark*

Department of Chemistry, Washington State University, Pullman, Washington 99164, United States; E-mail: zhu.liu@wsu.edu, auclark@wsu.edu

S1 Literature Analysis

Table S1: Previous theoretical and computational publications that have focused upon the water/octanol biphasic systems. Note that the works of Wipff and coworkers[1, 2, 3, 4] have examined metal-ligand complexe distributions across water/octanol interfaces as it pertains to liquid-liquid extraction. Computational protocols involved mixing/demixing (non-equilibrium) and potential of mean force were employed to understand the energetic favorability in the two bulk phases (where the distance coordinate is not the transport reaction coordinate).[5, 6] adopt mass-transfer models to understand the extraction equilibria and kinetics solute complexes between the aqueous and octanol phases.

References	partitioning coefficient (solute solubility, distribution coefficient)	interfacial structure	water solubility in octanol	water/solute transport mechanism
Campos-Villalobos2019[7]	✓	—	—	—
Qiao2018[8]	—	✓	✓	—
Cevc2018[9]	✓	✓	✓	—
Biswas2018Partitioning[10]	✓	✓	—	—
Schottl2018[11]	✓	✓	—	—
Zhang2018[12]	—	✓	✓	—
Biswas2018Alkali[13]	✓	✓	—	—
Lopez2018[14]	✓	—	—	—
Zolghadr2017[15]	✓	✓	—	—
Taddese2017[16]	✓	—	—	—
Mondal2017[17]	—	✓	—	—
Tang2017[18]	✓	—	—	—
Atsmon-Raz2017[19]	✓	—	—	—
Tabrizi2017[20]	✓	—	—	—
Saeedi2017[21]	✓	—	—	—
Singh2016[22]	✓	—	—	—
Bannan2016[23]	✓	—	—	—
Abranko-Rideg[24]	—	✓	—	—
Ndao[25]	—	—	✓	—
Schottl[26]	—	✓	—	—
Sun2015[27]	✓	—	—	—
Garrido2015[28]	✓	—	—	—
Wagner2015[29]	✓	—	—	—
Huang2015[30]	✓	—	—	—
Liyana-Arachchi2014[31]	✓	—	—	—
Wick2014[32]	—	✓	✓	—
Martin2014[33]	✓	—	—	—
Schottl2014[34]	—	✓	—	—
Liyana-Arachchi2013[35]	✓	—	—	—
Abranko-Rideg2013[36]	—	✓	—	—
Bhatnagar2013[37]	✓	—	—	—
Samanta2013[38]	✓	—	—	—

Kozbial2013[39]	✓	—	—	—
Benay2013[4]	✓	—	—	✓
Liyana-Arachchi2012[40]	✓	—	—	—
Samanta2012[41]	✓	—	—	—
Mohsen-Nia2012[42]	✓	—	—	—
Kamath2012[43]	✓	—	—	—
Bhatnagar2012[44]	✓	—	—	—
Park2011[45]	✓	—	—	—
Stein2011[46]	✓	—	—	—
Petersen2010[6]	✓	—	—	—
Palombo2010[47]	—	✓	—	—
Wick2010[48]	—	✓	—	—
Prasanna Rani2010[5]	✓	—	—	✓
Economou2010[49]	✓	—	—	—
Goudarzi2010[50]	✓	—	—	—
Benay2010[1]	—	—	—	✓
Noubigh2010[51]	✓	—	—	—
Garrido2009[52]	✓	—	—	—
Giri2009[53]	✓	—	—	—
Redmill2009[54]	✓	—	—	—
Carmosini2008[55]	✓	—	—	—
Nanzai2008[56]	✓	—	—	—
MacCallum2007[57]	✓	—	—	—
White2007[58]	✓	—	—	—
Chevrot2007[2]	—	—	—	✓
Cheng2007[59]	✓	—	—	—
Mintz2007[60]	✓	—	—	—
Held2007[61]	✓	—	—	—
Kazuya2007[62]	✓	—	—	—
Marrink2007[63]	✓	—	—	—
Ghasemi2007[64]	✓	—	—	—
Engelmann2007[65]	✓	—	—	—
Chevrot2007[3]	—	✓	—	✓
Jedlovszky2007[66]	—	✓	—	—
Chapeaux2007[67]	✓	—	—	—
Napoleon2006[68]	—	✓	—	—
Chen2006[69]	—	✓	—	—
Lamber2006[70]	✓	✓	—	—
Zhao2005[71]	✓	—	—	—
DeOliveira2005[72]	✓	—	—	—
Paolantoni2005[73]	—	✓	—	—
Jabusch2005[74]	✓	—	—	—
Meylan2005[75]	✓	—	—	—
Sassi2004[76]	—	✓	✓	—
Steel2004[77]	—	✓	—	—
Rabe2004[78]	✓	—	—	—
Benjamin2004[79]	—	✓	—	—
Jedlovszky2004[80]	—	✓	—	—
Zhang2003[81]	—	✓	—	—
Abraham2003[82]	✓	—	—	—
Steel2003[83]	—	✓	—	—
Aliste2003[84]	✓	✓	—	—
Bas2002[85]	✓	—	—	—
DeOliveira2002[86]	—	✓	—	—
Roberts2002[87]	✓	—	—	—
Kovalchuk200[88]	✓	—	—	—
Grassi2002[89]	✓	—	—	—
Peretti2002[90]	✓	—	—	✓
MacCallum2002[91]	—	✓	—	—
Tetko2001[92]	✓	—	—	—
Michael2001[93]	—	✓	—	—

Mulder2001[94]	✓	—	—	—
Curutchet2001[95]	✓	—	—	—
Nagy2000[96]	✓	—	—	—
Chen2000[97]	✓	—	—	—
Lin2000[98]	✓	—	—	—
Chimuka2000[99]	✓	—	—	—
Duffy2000[100]	✓	—	—	—
Pohorille1999[101]	—	✓	—	—
Michael1997[102]	—	✓	—	—

S2 Simulation Protocols/Models

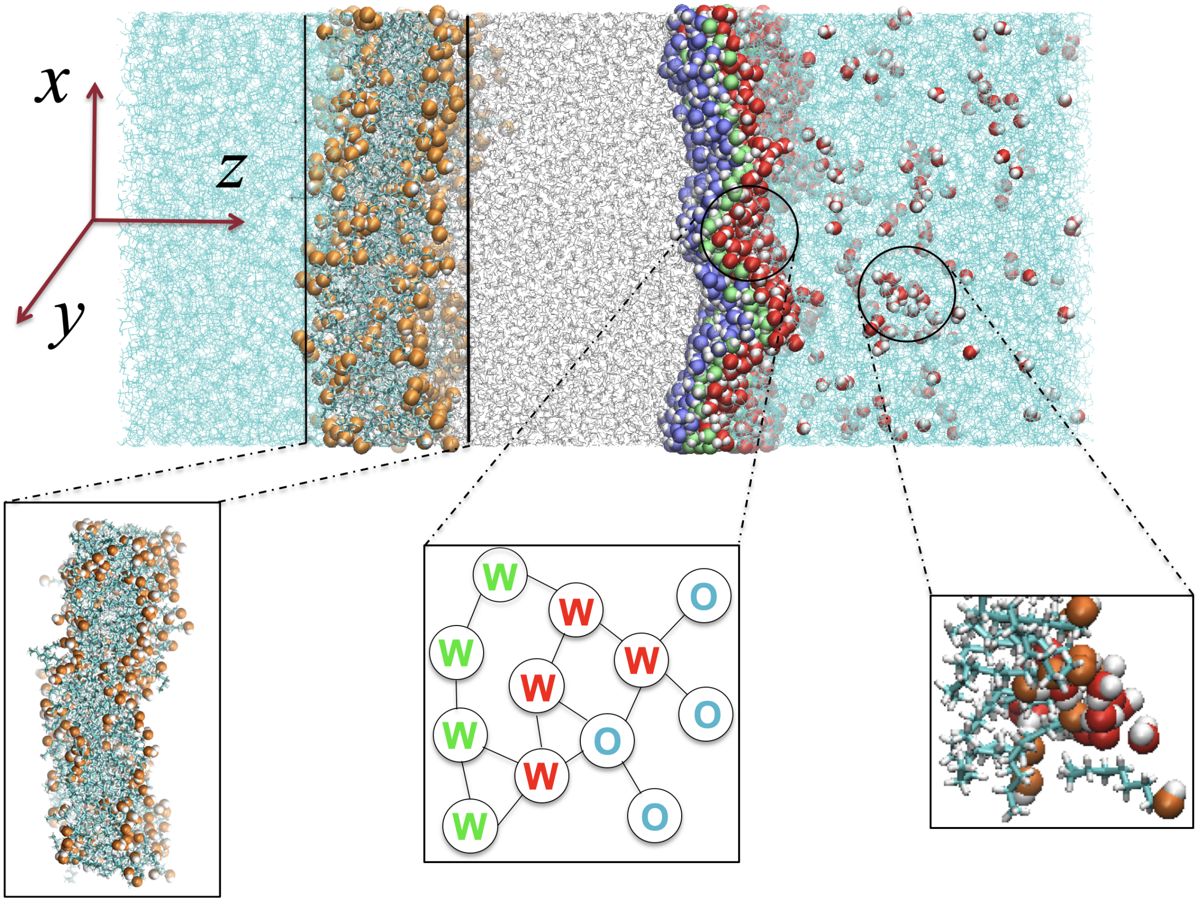

Figure S1: Schematic representation of the octanol/water liquid-liquid interface simulation box. Bulk water and octanol molecules are depicted in gray and cyan lines respectively. Extracted water molecules into the octanol phase and the interfacial water layer are described with red oxygens, while two adjacent water layers that directly beneath the interfacial water layer with green and blue oxygens, respectively (only shown in the right half cell). The bilayer structure of octanol molecules in the vicinity of the interface (left half box) is highlighted with cyan bonds for alkyl carbon tail and orange sphere for oxygens in the hydroxyl head group.

S2.1 Convergence of Simulation Data

Equilibrium is ascertained through monitoring a number of properties, including the interfacial tension, concentration of water in the octanol phase, and number of transport events between the two phases (*vide infra*)

Table S2: The compositions and simulation periodic unit cell box sizes, interfacial tensions, water solubilities after equlibration using the GROMOS54A7/SPC-E force field. Note that after 30 ns equilibration, system with 60 Å z length (40 Å of octanol phase along the z axis) is not sufficient enough to adequately describe the octanol long-range structure, as a consequence, no liquid-liquid interface is generated.

Water	Octanol	X	Y	Z	Interfacial Tension (mN/m)	Water fraction solubility
4279	979	77.96	77.96	60.42	—	—
8557	1959	78.24	78.24	119.32	7.89 ± 1.30	0.31
12836	2845	79.77	79.77	173.61	7.83 ± 0.80	0.26
17115	3917	78.54	78.54	237.59	7.43 ± 0.80	0.20
3209	734	40.60	40.60	166.92	7.44 ± 1.95	0.24
7220	1653	60.16	60.16	171.22	6.75 ± 0.65	0.25
20056	4591	98.16	98.16	178.64	7.76 ± 0.85	0.22

Table S3: Summary of calculated intermolecular interactions for water and octanol molecules with different octanol/water models. Here SR, Disper.-corr., and Coul.-recip. denote short-range, dispersion correction and coulombic interactions in reciprocal space.

Model	Intermoleular Energy Term		E (Octanol+Water)	E (Octanol)	E (Water)	Δ E (Octanol-Water)
GROMOS54A7/ SPCE	VDW	LJ (SR)	-3681.57±6.69	-30775.81±13.15	26710.08±10.04	384.16
		Disper. -corr.	-1473.01	-761.86	-116.03	-595.12
	Coulomb	Coulomb (SR)	-230431.40±9.80	-51595.84±20.32	-162230.88±28.68	-16604.68
		Coul.-recip.	1067.76±2.13	968.86±2.87	422.46±2.10	-323.56
GAFF/ TIP4P-Ew	VDW	LJ (SR)	-6768.52±5.26	-33036.57±8.37	26386.95±2.32	-118.90
		Disper. -corr.	-1691.97	-921.56	-116.02	-654.39
	Coulomb	Coulomb (SR)	-213773.42±12.91	-41068.83±14.34	-164735.66±5.26	-7968.93
		Coul.-recip.	672.33±0.79	492.45±0.57	329.73±0.38	-149.85
GAFF/ TIP3P	VDW	LJ (SR)	-21499.07±14.34	-39972.04±18.64	18552.15±3.82	-79.18
		Disper. -corr.	-1649.32	-920.30	-105.47	-623.55
	Coulomb	Coulomb (SR)	-189170.17±5.26	-44996.18±9.32	-134518.16±14.82	-9655.83
		Coul.-recip.	683.38±0.88	459.75±1.36	384.92±0.74	-161.29
OPLS/ TIP3P	VDW	LJ (SR)	-9892.33±4.54	-21459.39±3.82	11743.38±3.11	-176.32
		Disper. -corr.	-937.52	-504.49	-66.48	-366.55
	Coulomb	Coulomb (SR)	-93748.33±9.80	-2559.99±11.23	-87361.38±7.41	-3826.96
		Coul.-recip.	366.17±0.45	283.85±0.67	212.28±0.36	-129.96

S3 SI for the Results and Discussion

S3.1 Octanol interfacial structure in the z-direction

The black curve in the left panel of Figure S4 shows the scaled water density profile along the z axis by its bulk density in the octanol-rich phase. It can be observed that there presents a significant region of enhanced water concentration around 17 Å from the GDS toward the octanol phase, which is in good agreement with the previous simulation. The average densities of water and octanol more than 1 nm from the GDS in the octanol-rich phase yields an average mole fraction of water of 0.26, in good agreement with the experimental water solubility in octanol[103] (see Table 1).

The left panel of Figure S4 presents the density profiles of octanol atom types in the right half the simulation box along the z axis (scaled by their bulk densities in the octanol phase). The oscillation in the hydroxyl O-atom density profile draws the further interest, with its first peak sitting exactly at the position of the Gibbs Dividing surface (GDS) (z = 0). The density has a minimum near 9 Å from the GDS, which is in good agreement with previous simulations having a similar distances of 8[79] or 9 Å[32]. We further calculate the average octanol molecular end-to-end distance (hydroxyl oxygen to methyl carbon) to be 8.68 Å, coinciding with the difference between the first maximum and minimum of the hydroxyl oxygen density profile. The density oscillation propagates further along the z dimension of the simulation box, with the second peak sitting ∼17 Å from the GDS, a value comparable to twice the octanol molecular end-to-end length. The average end-to-end distance of octanol molecules in the second layer of the bilayer structure has a value is 8.54 Å. It is worth to note that the sum of this obtained value and the average interfacial octanol molecular

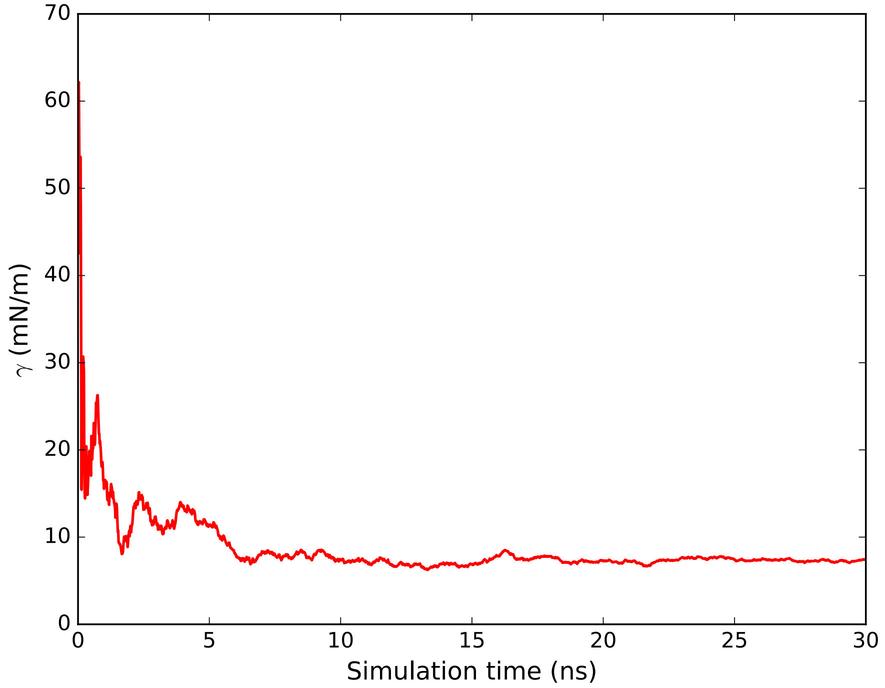

Figure S2: Convergence of interfacial tension, as calculated using Equation 1 in the main text, as a function of equilibration time.

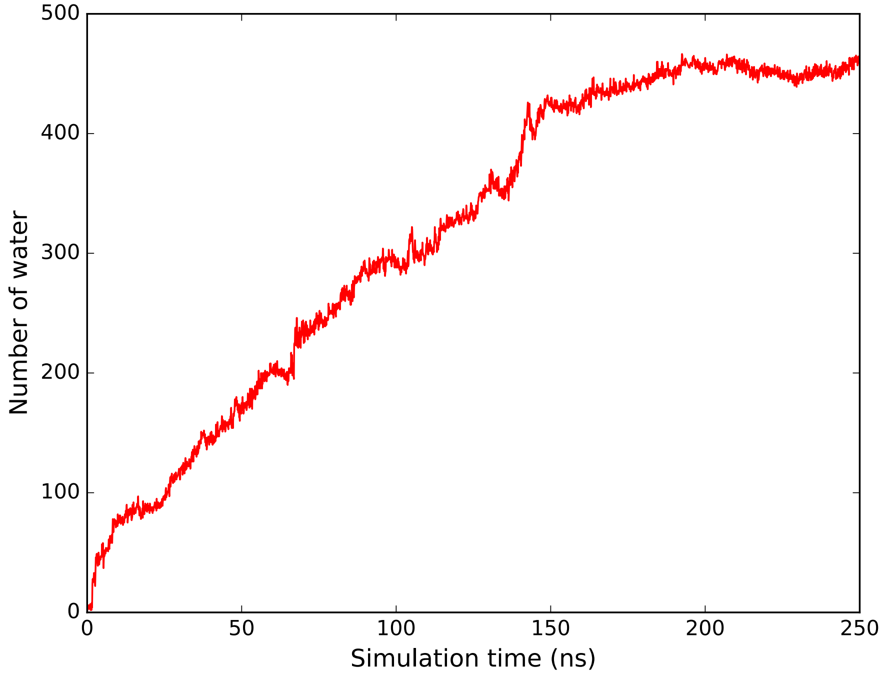

Figure S3: Simulated number of water molecules in octanol phase as a function of simulation time.

end-to-end distance is confirmed to be equal to the difference between the aboved-mentioned maxima of the hydroxyl oxygen density profile. The distribution of the molecular end-to-end distance of the octanol backbone

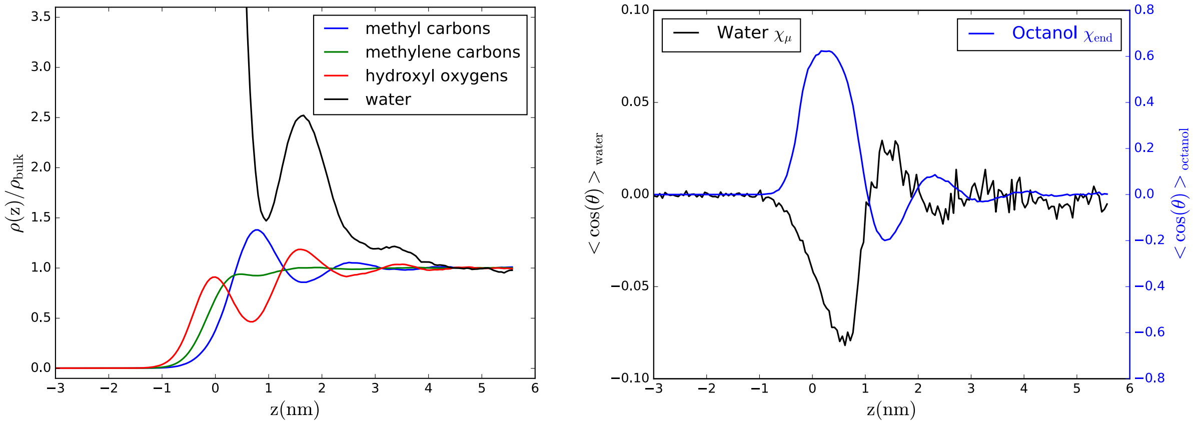

Figure S4: Scaled density profiles from the octanol-water liquid-liquid interface system for water (black line), octanol hydroxy oxygens (red line), octanol methylene carbons (green line), and octanol methyl carbons (blue line) (left panel). Average orientation of water dipoles (black lines) and end-to-end atoms (hydrogen from OH groups and methyl carbon) for octanol (blue lines). The angle is with respect to the z axis, with zero representing no orientational preference for both profiles (right panel).

(hydroxyl oxygen to methyl carbon) in Layer-1 and Layer-2 are shown in Figure S5.

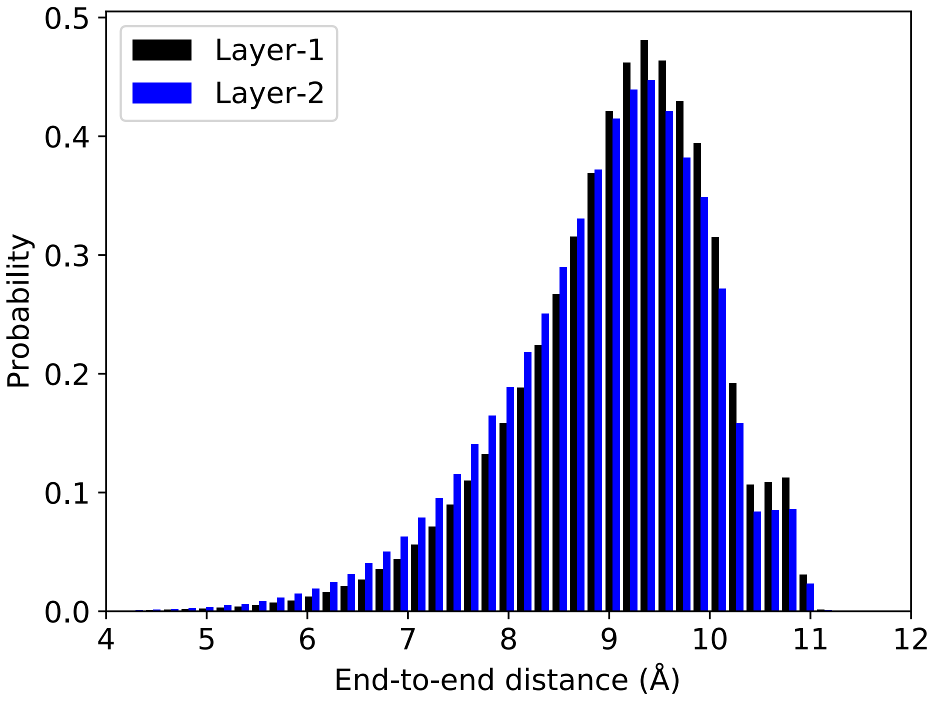

Figure S5: The distribution of the molecular end-to-end distance for octanol in Layer-1 and Layer-2.

S3.2 Octanol structure parallel to the interface

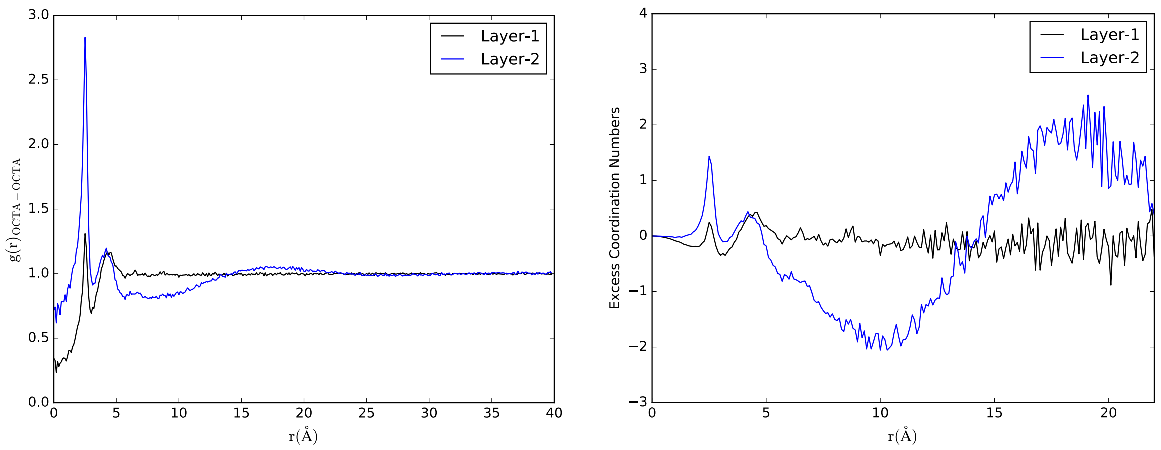

Figure S6: Left panel: Two dimensional RDF of bilayer octanol molecules (O...O) in the water-octanol interface system. Right panel: Excess coordination numbers between octanol molecules in Layer-1 and Layer-2.

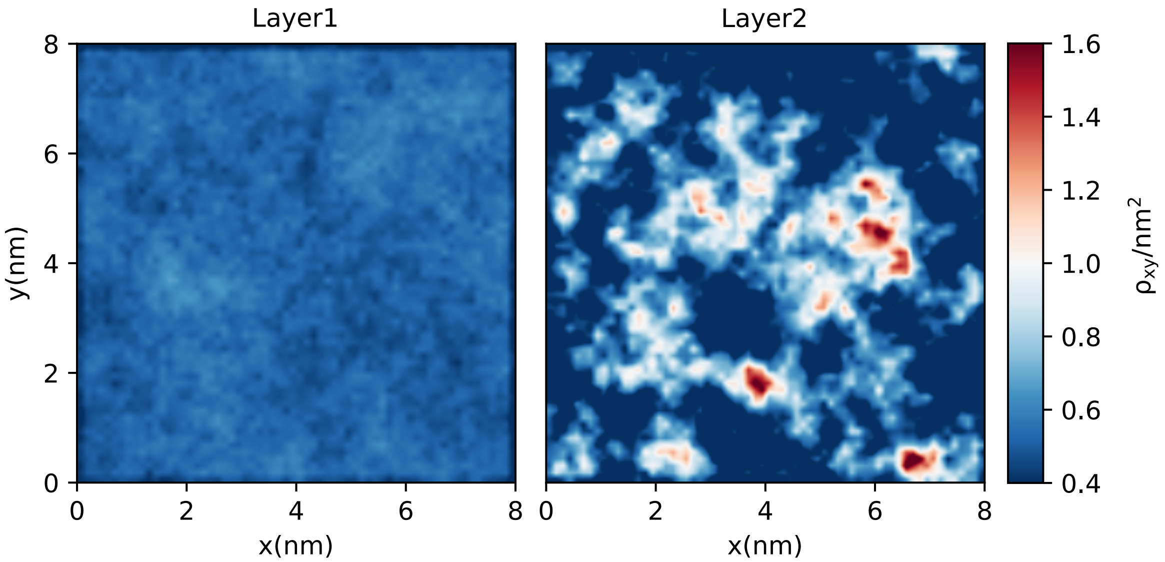

Figure S7: Two-dimensional normed density distributions of water molecules that are hydrogen bonded to octanol molecules in Layer-1 and Layer-2 with 1.0 representing the bulk uniform distribution.

Table S4: The dataset of compositions of islands formed by water and octanol hydroxyl atoms in the Layer-2 for all examined 3000 frames of simulation data.

Composition	0water	1water	2water	3water	4water	5water	6water	7water	8water	9water	10water	11water	12water	13water	14water	15water
0octanol	—	0	2	1	0	0	0	0	0	0	0	0	0	0	0	0
1octanol	0	64	44	23	6	3	0	0	0	0	0	0	0	0	0	0
2octanol	271	206	208	416	153	24	3	0	0	0	0	0	0	0	0	0
3octanol	433	639	2191	1347	267	64	11	1	1	0	0	0	0	0	0	0
4octanol	891	6029	4366	1556	501	134	35	7	0	0	0	0	0	0	0	0
5octanol	5880	7539	3740	1854	755	202	68	23	2	2	1	0	0	0	0	0
6octanol	4186	4846	3304	1879	899	328	109	41	23	5	1	1	0	0	0	0
7octanol	1775	2806	2486	1657	877	446	195	65	37	10	5	4	0	0	0	0
8octanol	753	1449	1590	1404	800	500	251	95	38	35	7	6	3	0	0	0
9octanol	288	670	959	938	731	472	268	107	66	33	13	7	2	0	0	0
10octanol	104	291	528	631	545	378	251	142	69	27	24	8	4	5	1	1
11octanol	38	123	249	376	375	305	217	134	66	42	24	12	4	1	2	1
12octanol	15	60	134	267	227	212	184	113	48	41	19	15	4	2	1	0
13octanol	7	26	76	104	144	145	123	106	53	43	23	14	5	0	2	1
14octanol	1	9	27	57	94	91	84	75	52	23	15	17	5	5	4	1
15octanol	0	3	13	34	59	73	58	62	48	35	17	8	6	2	4	0
16octanol	0	1	7	13	38	32	43	29	34	13	11	8	6	0	4	1
17octanol	0	2	3	9	12	19	28	33	30	12	14	8	5	4	2	0
18octanol	0	1	1	3	6	7	17	17	20	11	13	9	5	2	2	1
19octanol	0	1	0	2	6	3	7	10	3	10	15	8	6	1	0	0
20octanol	0	0	1	1	3	3	3	12	8	9	11	6	3	1	1	0
21octanol	0	0	0	1	1	1	4	0	3	6	3	6	0	1	0	0
22octanol	0	0	0	0	0	0	0	4	2	2	4	3	0	0	0	0
23octanol	0	0	0	0	0	0	0	0	1	1	2	2	1	0	1	0
24octanol	0	0	0	0	0	0	0	0	2	0	1	2	0	0	1	1
25octanol	0	0	0	0	0	0	0	2	0	1	1	0	2	0	0	0
26octanol	0	0	0	0	0	1	0	0	0	0	0	0	0	0	0	0

S3.3 Water and octanol transport dynamics.

The observed transport events of water and octanol across Layer-1 $\Longleftrightarrow$ Layer-2 and Layer-2 $\Longleftrightarrow$ organic phase are first presented, with subsequent tables illustrating all terms in the Arrhenius determination of E_a for transport.

S3.3.1 Oberved Transport Events and Molecular Composition.

Table S5: Observed transfer events of octanol and water-octanol clusters ("molecular hinges") for Layer-1 $\Longleftrightarrow$ Layer-2, using 10 ps sampling. The stochastic flipping of individual octanol molecules is identified and separated from diffusion water transport events and those that involve a cluster of $(H_2O)_n(\mathrm{oct})_m$.

**Stochastic Octanol Flipping**	Layer-1 → Layer-2	Layer-2 → Layer-1
1octanol	13269	13024
2octanol	128	135
3octanol	21	26
4octanol	1	2
5octanol	1	–
Sum	13420	13187
**Diffusion Water Transport**		
1water	477	413
2water	–	1
Sum	447	413
**Stochastic Water Transport**		
1Water·1Octanol	168	182
**Water Transport by Hinge Mechanism**		
1Water·2Octanol	293	310
1Water·3Octanol	243	231
1Water·4Octanol	67	81
1Water·5Octanol	14	11
2Water·1Octanol	3	3
2Water·2Octanol	39	35
2Water·3Octanol	84	101
2Water·4Octanol	88	79
2Water·5Octanol	44	38
2Water·6Octanol	12	12
2Water·7Octanol	–	1
2Water·8Octanol	–	1
3Water·1Octanol	–	1

3Water·2Octanol	2	4
3Water·3Octanol	23	19
3Water·4Octanol	42	37
3Water·5Octanol	31	40
3Water·6Octanol	16	22
3Water·7Octanol	8	3
3Water·8Octanol	1	–
3Water·9Octanol	1	–
4Water·1Octanol	–	1
4Water·2Octanol	1	1
4Water·3Octanol	4	4
4Water·4Octanol	10	14
4Water·5Octanol	18	25
4Water·6Octanol	28	13
4Water·7Octanol	5	5
4Water·8Octanol	7	2
5Water·2Octanol	–	1
5Water·3Octanol	–	2
5Water·4Octanol	9	5
5Water·5Octanol	7	5
5Water·6Octanol	15	13
5Water·7Octanol	12	8
5Water·8Octanol	5	7
5Water·9Octanol	4	3
6Water·2Octanol	–	1
6Water·4Octanol	2	1
6Water·5Octanol	5	4
6Water·6Octanol	5	5
6Water·7Octanol	5	13
6Water·8Octanol	8	5
6Water·9Octanol	1	6
7Water·4Octanol	1	2
7Water·5Octanol	3	1
7Water·6Octanol	2	–
7Water·7Octanol	4	2
7Water·8Octanol	4	4
7Water·9Octanol	3	4
7Water·10Octanol	2	1
7Water·11Octanol	–	1
8Water·4Octanol	–	2
8Water·5Octanol	3	–
8Water·6Octanol	1	1
8Water·7Octanol	1	2
8Water·8Octanol	3	7
8Water·9Octanol	3	4
8Water·10Octanol	3	2
8Water·11Octanol	2	–
8Water·12Octanol	1	–
9Water·6Octanol	1	–
9Water·7Octanol	2	1
9Water·8Octanol	–	2
9Water·9Octanol	3	–
9Water·10Octanol	1	–
10Water·7Octanol	1	1
10Water·8Octanol	3	2
10Water·9Octanol	1	–
10Water·11Octanol	1	–
10Water·12Octanol	1	–
11Water·6Octanol	–	1
11Water·7Octanol	3	1
11Water·8Octanol	2	1
11Water·9Octanol	1	–
11Water·11Octanol	2	1
12Water·9Octanol	2	–
12Water·10Octanol	–	1
12Water·13Octanol	1	2
13Water·10Octanol	–	1
14Water·10Octanol	–	1
14Water·11Octanol	–	1
Sum of Events	1223	1221

Table S6: Observed transfer events of octanol and water-octanol clusters migrating from Layer-2 to bulk octanol. Sums of all transport events are noted.

Composition	Layer-2 → Organic	Organic → Layer-2
1octanol	79248	79165
2octanol	1399	1529
3octanol	406	448
4octanol	68	57
5octanol	12	16
6octanol	2	4
7octanol	–	2
Sum of Events	81135	81221
1water	18554	18573
2water	201	202
3water	46	50
4water	9	11
Sum of Events	18810	18836
1Water·1Octanol	2640	2716
1Water·2Octanol	1332	1191
1Water·3Octanol	233	226
1Water·4Octanol	44	35
1Water·5Octanol	13	10
1Water·6Octanol	2	4
1Water·7Octanol	1	1
1Water·8Octanol	1	–
2Water·1Octanol	1009	975
2Water·2Octanol	386	385
2Water·3Octanol	69	94
2Water·4Octanol	31	22
2Water·5Octanol	10	7
2Water·6Octanol	3	1
2Water·7Octanol	1	1
3Water·1Octanol	149	149
3Water·2Octanol	72	93
3Water·3Octanol	34	30
3Water·4Octanol	12	4
3Water·5Octanol	4	2
3Water·6Octanol	1	2
4Water·1Octanol	18	16
4Water·2Octanol	24	21
4Water·3Octanol	10	4
4Water·4Octanol	3	2
4Water·5Octanol	–	1
4Water·6Octanol	–	1
4Water·7Octanol	1	–
4Water·9Octanol	–	1
5Water·1Octanol	2	3
5Water·2Octanol	3	7
5Water·3Octanol	4	1
5Water·4Octanol	2	–
5Water·5Octanol	–	1
6Water·1Octanol	1	–
7Water·4Octanol	1	–
Sum of Events	6116	6006

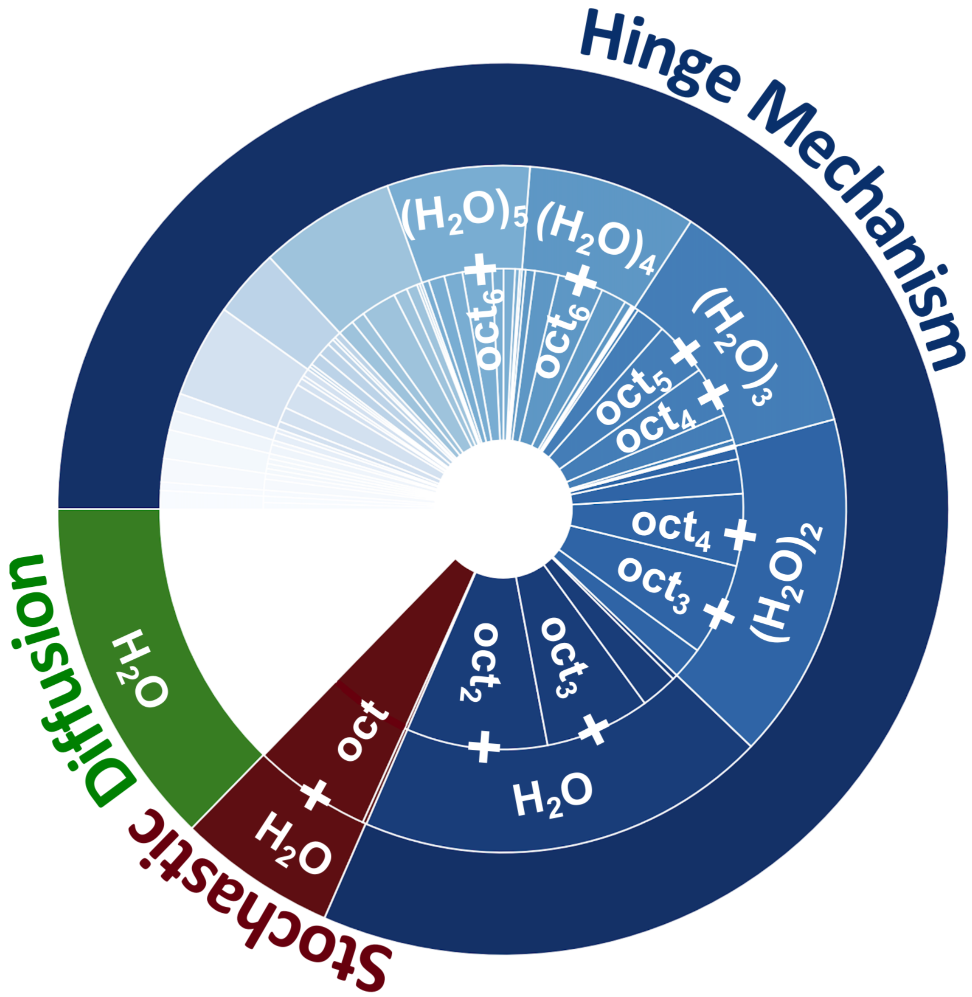

Figure S8: The relative contributions of different water Layer-2 → Layer-1 transport mechanisms (diffusion, stochastic octanol flipping, and the hinge mechanism) observed within the water/octanol system, and the composition of transporting species within each transport mechanism. Water diffusion comprises 14% of all transported water (in green). while 6% are transported by a single hydrogen bond to a stochastically flipping single octanol (in red), and 80% are transported by an octanol molecular hinge that is composed of $(\text{oct})_n(\text{H}_2\text{O})_m$ clusters (in blue). The relative contributions of each cluster composition is provided within the inner rings of the plot. Only transport species with a percentage above 5% are listed.

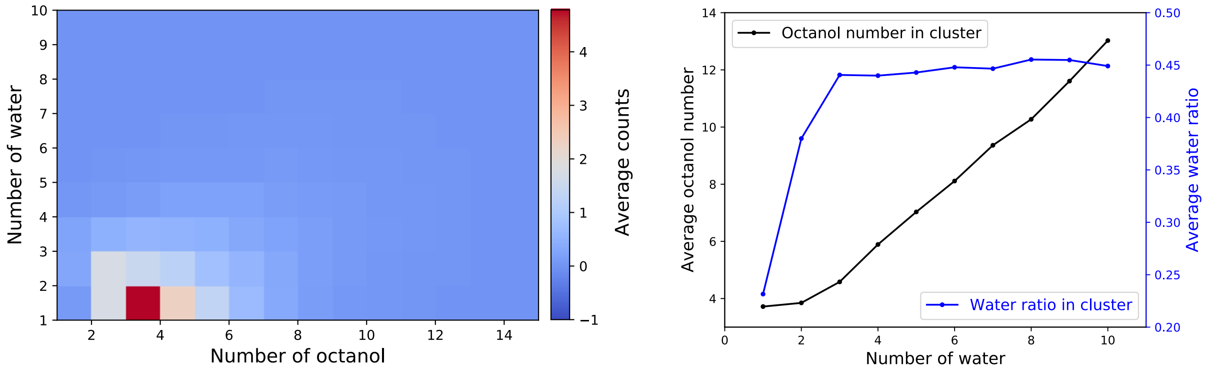

Figure S9: Left panel: The average distribution of the composition of clusters formed by water and octanol hydroxyl atoms in the Layer-2. Right panel: Correlation of water and octanol molecules in the composition of clusters. (The used data are listed in the Table S4)

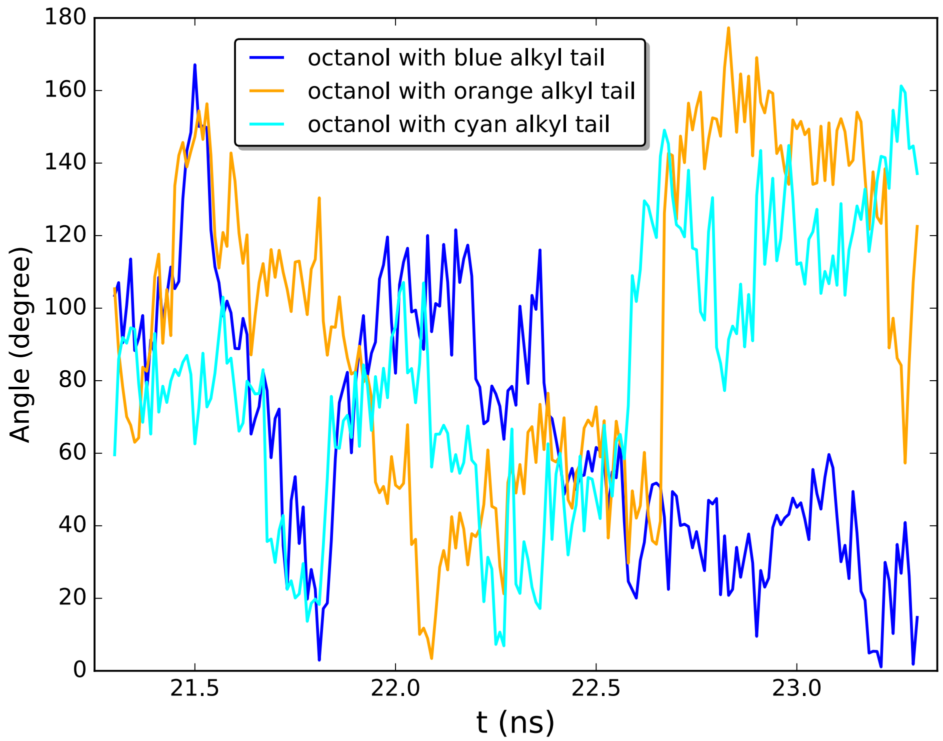

Figure S10: Orientation profiles of the octanol molecular machine in the process of transporting water molecules between Layer-1 and Layer-2. The angle is between the octanol molecular end-to-end atoms (hydroxyl hydrogen and methyl carbon) and the z axis (see Figure S1). The color code is described in Figure 3.

S3.3.2 Determining E_a for Transport.

The stochastic flipping of individual octanol between Layer-1 $\Longleftrightarrow$ Layer-2 was first examined. For A, we presume that in order for an octanol to stochastically flip, it must not be hydrogen bonded to any other species, and thus A is estimated to be the rate at which octanol within a layer loses its hydrogen bonds to other octanol and H_2O.

Table S7: Arrhenius equation fitting data for the stochastic unimolecular octanol flipping Layer-1 $\Longleftrightarrow$ Layer-2. Transport rates are in /10 ps and concentration is in number of molecules. Prefactor A was estimated based upon the rate at which octanol within a layer lose all hydrogen bonds to other octanol or waters.

Oct Layer-1 $\longrightarrow$ Layer-2					
E_a (kcal/mol)	oct flipping rate	$[oct]_{L1}$	Rxn Order	k	A
0.25±0.01	1308.71±52.39	484.03±2.56	1	2.70±0.09	4.11±0.11
Oct Layer-2 $\longrightarrow$ Layer-1					
E_a (kcal/mol)	oct flipping rate	$[oct]_{L2}$	Rxn Order	k	A
0.21±0.02	1309.18±53.52	427.67±3.83	1	3.06±0.10	4.33±0.01

On an average 3.71 ± 0.05 octanol molecules are involved in the Layer-1 → Layer-2 transfer of 2.28 ± 0.11 H_2O per transfer event, while 3.65 ± 0.05 octanol molecules participate in Layer-1 ← Layer-2 transfer of 2.24 ± 0.07 H_2O per transfer event. Based upon the compositions of all transferring clusters of octanol and water (Layer-1 $\Longleftrightarrow$ Layer-2), the overall reaction for transport only of a single water using the hinge mechanism is:

Layer-1 $H_2O(oct)_{1.63\pm0.08}$ $\Longleftrightarrow$ Layer-2 $H_2O(oct)_{1.63\pm0.03}$

To be able to employ the Arrhenius equation using this chemical reaction is however not possible, as to determine the rate constant, the both the rate of transfer and the total concentration of all reactant and products is needed. Within the analyses performed only the successful transfer processes are observed, as such the total concentration is not known.

Instead, the Arrhenius equation is employed for the following reaction:

Layer-1 H_2O + 1.63oct $\Longleftrightarrow$ Layer-2 H_2O + 1.63oct

This reaction encompasses both the formation of the $H_2O(oct)_{1.63}$ reactive hinge structures from all water and octanols in the layer to *and* the transport of water in the hinge form. In this case, the concentrations of all reactant and product species are known.

Two estimates of the prefactor A have been employed: 1) using the rate of new hydrogen bond formation between water-octanol (in that a water-octanol cluster cannot form without the formation of a HB), or 2) the rate of new waters that adsorb to the surface and hydrogen bond with octanol in Layer-1 and Layer-2. The complete table of all values and associated calculated E_a values with all statistical uncertainties is presented below, employing the following equation:

$$E_a = -ln(k/A) * RT \tag{1}$$

Using the predicted E_a values and the associated statistical errors, a series of Langevin Dynamic simulations were performed using a double well potential, as described in the Computational Methods section. The Table below presents the fitted parameters of the double well analytical representation for the different respective E_a values (and those including statistical uncertainties), along with the equilibrated ratio of particles in the left-hand (Layer-1) side and right-hand (Layer-2) side of the well potential.

Table S8: Arrhenius equation fitting data for the molecular transport in the process of water transport between Layer-1 $\Longleftrightarrow$ Layer-2. Transport rates are in /10 ps and concentration is in number of molecules. Prefactor A was estimated based upon two different rates: (a) the rate of new hydrogen bond formation in Layer-1 or Layer-2, and (b) the rate of new water adsorption into Layer-1 or Layer-2.

Layer-1 $\rightarrow$ Layer-2						
E_a (kcal/mol)	H_2O trans. rate	$[H_2O]_{L1}$	$[oct]_{L1}$	Rxn Order	k	A
6.24±0.24	0.92±0.06	927.07±4.89	484.03±2.56	2.63±0.08	4.53E-08±1.88E-08	1.51E-03±2.24E-05[a]
5.93±0.25	0.92±0.06	927.07±4.89	484.03±2.56	2.63±0.08	4.53E-08±1.88E-08	9.03E-03±5.76E-06[b]
Layer-2 $\rightarrow$ Layer-1						
E_a (kcal/mol)	H_2O trans. rate	$[H_2O]_{L2}$	$[oct]_{L2}$	Rxn Order	k	A
5.48±0.12	0.95±0.04	87.90±3.87	427.67±3.83	2.63±0.03	5.60E-07±1.21E-07	5.45E-03±8.34E-05[a]
4.33±0.11	0.95±0.04	87.90±3.87	427.67±3.83	2.62±0.03	5.60E-07±1.21E-07	7.86E-04±2.47E-05[b]

Table S9: Results from Langevin dynamics, where the double-well potential function U, the respective E_a values for Layer-1 $\rightarrow$ Layer-2, and E_a for Layer-2 $\rightarrow$ Layer-1, and the ratio (R $L1/L2$) of transferred particles are presented.

U	E_a L1→L2	E_a L2→L1	R ($L1/L2$)
$9.5892z^4-0.22z^3-15.0z^2+0.6052z+6.24+0.5x^2$	6.24	5.24	1.31±0.15
$9.6549z^4-0.22z^3-14.9699z^2+0.3982z+6.0+0.5x^2$	6.24-0.24	5.48+0.12	1.16±0.11
$9.8918z^4-0.22z^3-15.4709z^2+0.6713z+6.48+0.5x^2$	6.24+.24	5.48+0.12	1.37±0.14
$9.8477z^4-0.22z^3-14.9599z^2+0.5331z+6.0+0.5x^2$	6.24-0.24	5.48-0.12	1.28±0.12
$9.2986z^4-0.22z^3-13.8116z^2+1.0892z+5.93+0.5x^2$	5.93	4.33	1.72±0.17
$9.47495z^4-0.22z^3-13.8557z^2+0.87876z+5.68+0.5x^2$	5.93-0.25	4.33+0.11	1.51±0.12
$9.5170z^4-0.22z^3-14.21810z^2+1.1673z+6.18+0.5x^2$	5.93+0.25	4.33+0.11	1.84±0.17
$9.8477z^4-0.22z^3-13.9880z^2+1.0080z+5.68+0.5x^2$	5.93-0.25	4.33-0.11	1.68±0.14

References

[1] G. Benay, R. Schurhammer, and G. Wipff. BTP-based ligands and their complexes with Eu3+ at "oil"/water interfaces. A molecular dynamics study. *Physical Chemistry Chemical Physics*, 12(36):11089–11102, 2010.

[2] G. Chevrot, R. Schurhammer, and G. Wipff. Molecular dynamics study of dicarbollide anions in nitrobenzene solution and at its aqueous interface. Synergistic effect in the Eu(III) assisted extraction. *Physical Chemistry Chemical Physics*, 9(44):5928–5938, 2007.

[3] G Chevrot, R Schurhammer, and G Wipff. Synergistic effect of dicarbollide anions in liquid–liquid extraction: a molecular dynamics study at the octanol–water interface. *Physical Chemistry Chemical Physics*, 9(16):1991–2003, 2007.

[4] G. Benay and G. Wipff. Oil-soluble and water-soluble BTPhens and their europium complexes in octanol/water solutions: Interface crossing studied by MD and PMF simulations. *Journal of Physical Chemistry B*, 117(4):1110–1122, 2013.

[5] K. N. Prasanna Rani, T. Prathap Kumar, J. S.N. Murthy, T. Sankarshana, and B. Vishwanadham. Equilibria, Kinetics, and Modeling of Extraction of Citric Acid from Aqueous Solutions with Alamine 336 in 1-Octanol. *Separation Science and Technology*, 45(5):654–662, 2010.

[6] Elijah J. Petersen, Qingguo Huang, and Walter J. Weber. Relevance of octanol-water distribution measurements to the potential ecological uptake of multi-walled carbon nanotubes. *Environmental Toxicology and Chemistry*, 29(5):1106–1112, 2010.

[7] Gerardo Campos-Villalobos, Flor R Siperstein, and Alessandro Patti. Transferable coarse-grained martini model for methacrylate-based copolymers. *Molecular Systems Design & Engineering*, 4(1):186–198, 2019.

[8] Baofu Qiao and Wei Jiang. All-Atom Molecular Dynamics Study of Water-Dodecane Interface in the Presence of Octanol. *Journal of Physical Chemistry C*, 122(1):687–693, 2018.

[9] Gregor Cevc, Ida Berts, Stefan F. Fischer, Joachim O. Rädler, and Bert Nickel. Nanostructures in n-Octanol Equilibrated with Additives and/or Water. *Langmuir*, 34(21):6285–6295, 2018.

[10] Rima Biswas, Pallab Ghosh, Tamal Banerjee, and Sk Musharaf Ali. Partitioning of Cs+ and Na+ ions by dibenzo-18-crown-6 ionophore in biphasic aqueous systems of octanol and ionic liquid. *Radiochimica Acta*, 106(6):477–495, 2018.

[11] Sebastian Schöttl and Dominik Horinek. Salt effects in surfactant-free microemulsions. *Journal of Chemical Physics*, 148(22):1–6, 2018.

[12] Hao Zhang, Wengang Liu, Cong Han, and Dezhou Wei. Intensify dodecylamine adsorption on magnesite and dolomite surfaces by monohydric alcohols. *Applied Surface Science*, 444:729–738, 2018.

[13] Rima Biswas, Abhigyan Malviya, Tamal Banerjee, Pallab Ghosh, and Sk Musharaf Ali. Alkali Metal Ion Partitioning with Calix[4]arene-benzo-crown-6 Ionophore in Acidic Medium: Insights from Experiments, Statistical Mechanical Framework, and Molecular Dynamics Simulations. *Journal of Physical Chemistry B*, 122(7):2102–2112, 2018.

[14] Cesar A. López, Clifford J. Unkefer, Basil I. Swanson, Jessica M.J. Swanson, and S. Gnanakaran. Membrane perturbing properties of toxin mycolactone from Mycobacterium ulcerans. *PLoS Computational Biology*, 14(2):1–22, 2018.

[15] Amin Reza Zolghadr and Samaneh Boroomand. Spontaneous assembly of HSP90 inhibitors at water/octanol interface: A molecular dynamics simulation study. *Chemical Physics Letters*, 669:130–136, 2017.

[16] Tseden Taddese and Paola Carbone. Effect of Chain Length on the Partition Properties of Poly(ethylene oxide): Comparison between MARTINI Coarse-Grained and Atomistic Models. *Journal of Physical Chemistry B*, 121(7):1601–1609, 2017.

[17] Jahur A. Mondal, V. Namboodiri, P. Mathi, and Ajay K. Singh. Alkyl Chain Length Dependent Structural and Orientational Transformations of Water at Alcohol-Water Interfaces and Its Relevance to Atmospheric Aerosols. *Journal of Physical Chemistry Letters*, 8(7):1637–1644, 2017.

[18] Xueming Tang, Weizhong Zou, Peter H. Koenig, Shawn D. McConaughy, Mike R. Weaver, David M. Eike, Michael J. Schmidt, and Ronald G. Larson. Multiscale Modeling of the Effects of Salt and Perfume Raw Materials on the Rheological Properties of Commercial Threadlike Micellar Solutions. *Journal of Physical Chemistry B*, 121(11):2468–2485, 2017.

[19] Yoav Atsmon-Raz and D. Peter Tieleman. Parameterization of Palmitoylated Cysteine, Farnesylated Cysteine, Geranylgeranylated Cysteine, and Myristoylated Glycine for the Martini Force Field. *Journal of Physical Chemistry B*, 121(49):11132–11143, 2017.

[20] Amirhossein Molavi Tabrizi, Spencer Goossens, Ali Mehdizadeh Rahimi, Christopher D. Cooper, Matthew G. Knepley, and Jaydeep P. Bardhan. Extending the Solvation-Layer Interface Condition Continum Electrostatic Model to a Linearized Poisson-Boltzmann Solvent. *Journal of Chemical Theory and Computation*, 13(6):2897–2914, 2017.

[21] Marzieh Saeedi, Alexander P. Lyubartsev, and Seifollah Jalili. Anesthetics mechanism on a DMPC lipid membrane model: Insights from molecular dynamics simulations. *Biophysical Chemistry*, 226:1–13, 2017.

[22] Moirangthem Kiran Singh, Him Shweta, Mohammad Firoz Khan, and Sobhan Sen. New insight into probe-location dependent polarity and hydration at lipid/water interfaces: Comparison between gel- and fluid-phases of lipid bilayers. *Physical Chemistry Chemical Physics*, 18(35):24185–24197, 2016.

[23] Caitlin C. Bannan, Gaetano Calabró, Daisy Y. Kyu, and David L. Mobley. Calculating Partition Coefficients of Small Molecules in Octanol/Water and Cyclohexane/Water. *Journal of Chemical Theory and Computation*, 12(8):4015–4024, 2016.

[24] Nóra Abrankó-Rideg, George Horvai, and Pál Jedlovszky. Structure of the adsorption layer of various ionic and non-ionic surfactants at the free water surface, as seen from computer simulation and ITIM analysis. *Journal of Molecular Liquids*, 205:9–15, 2015.

[25] Makha Ndao, Julien Devemy, Aziz Ghoufi, and Patrice Malfreyt. Coarse-graining the liquid–liquid interfaces with the martini force field: How is the interfacial tension reproduced? *Journal of Chemical Theory and Computation*, 11(8):3818–3828, 2015.

[26] Sebastian Schöttl, Didier Touraud, Werner Kunz, Thomas Zemb, and Dominik Horinek. Consistent definitions of "the interface" in surfactant-free micellar aggregates. *Colloids and Surfaces A: Physicochemical and Engineering Aspects*, 480:222–227, 2015.

[27] Delin Sun, Jan Forsman, and Clifford E Woodward. Evaluating force fields for the computational prediction of ionized arginine and lysine side-chains partitioning into lipid bilayers and octanol. *Journal of Chemical Theory and Computation*, 11(4):1775–1791, 2015.

[28] Nuno M Garrido, J Queimada, Miguel Jorge, and A Macedo. Pyrolysis of Heavy Oil in the Presence of Supercritical Water: The Reaction Kinetics in Different Phases. *AICHE Journal*, 61(3):857–866, 2015.

[29] Bjoern Wagner, Holger Fischer, Manfred Kansy, Anna Seelig, and Frauke Assmus. Carrier Mediated Distribution System (CAMDIS): A new approach for the measurement of octanol/water distribution coefficients. *European Journal of Pharmaceutical Sciences*, 68:68–77, 2015.

[30] Wenjuan Huang, Nikolay Blinov, and Andriy Kovalenko. Octanol-Water Partition Coefficient from 3D-RISM-KH Molecular Theory of Solvation with Partial Molar Volume Correction. *Journal of Physical Chemistry B*, 119(17):5588–5597, 2015.

[31] Thilanga P. Liyana-Arachchi, Zenghui Zhang, Harsha Vempati, Amie K. Hansel, Christopher Stevens, Andrew T. Pham, Franz S. Ehrenhauser, Kalliat T. Valsaraj, and Francisco R. Hung. Green leaf volatiles on atmospheric air/water interfaces: A combined experimental and molecular simulation study. *Journal of Chemical and Engineering Data*, 59(10):3025–3035, 2014.

[32] Collin D Wick and Tsun-Mei Chang. Computational observation of pockets of enhanced water concentration at the 1-octanol/water interface. *The Journal of Physical Chemistry B*, 118(28):7785–7791, 2014.

[33] Lewis J. Martin, Rebecca Chao, and Ben Corry. Molecular dynamics simulation of the partitioning of benzocaine and phenytoin into a lipid bilayer. *Biophysical Chemistry*, 185:98–107, 2014.

[34] S. Schöttl, J. Marcus, O. Diat, D. Touraud, W. Kunz, T. Zemb, and D. Horinek. Emergence of surfactant-free micelles from ternary solutions. *Chemical Science*, 5(8):2949–2954, 2014.

[35] Thilanga P. Liyana-Arachchi, Amie K. Hansel, Christopher Stevens, Franz S. Ehrenhauser, Kalliat T. Valsaraj, and Francisco R. Hung. Molecular modeling of the green leaf volatile methyl salicylate on atmospheric air/water interfaces. *Journal of Physical Chemistry A*, 117(21):4436–4443, 2013.

[36] Nóra Abrankó-Rideg, Mária Darvas, George Horvai, and Pál Jedlovszky. Immersion depth of surfactants at the free water surface: A computer simulation and ITIM analysis study. *Journal of Physical Chemistry B*, 117(29):8733–8746, 2013.

[37] Navendu Bhatnagar, Ganesh Kamath, and Jeffrey J. Potoff. Prediction of 1-octanol-water and air-water partition coefficients for nitro-aromatic compounds from molecular dynamics simulations. *Physical Chemistry Chemical Physics*, 15(17):6467–6474, 2013.

[38] Susruta Samanta, Samira Hezaveh, and Danilo Roccatano. Theoretical study of binding and permeation of ether-based polymers through interfaces. *Journal of Physical Chemistry B*, 117(47):14723–14731, 2013.

[39] Małgorzata Koźbiał and Paweł Gierycz. Comparison of aqueous and 1-octanol solubility as well as liquid-liquid distribution of acyclovir derivatives and their complexes with hydroxypropyl-β-cyclodextrin. *Journal of Solution Chemistry*, 42(4):866–881, 2013.

[40] Thilanga P. Liyana-Arachchi, Kalliat T. Valsaraj, and Francisco R. Hung. Adsorption of naphthalene and ozone on atmospheric air/ice interfaces coated with surfactants: A molecular simulation study. *Journal of Physical Chemistry A*, 116(10):2519–2528, 2012.

[41] Susruta Samanta, Samira Hezaveh, Giuseppe Milano, and Danilo Roccatano. Diffusion of 1,2-dimethoxyethane and 1,2-dimethoxypropane through phosphatidycholine bilayers: A molecular dynamics study. *Journal of Physical Chemistry B*, 116(17):5141–5151, 2012.

[42] M. Mohsen-Nia, A. H. Ebrahimabadi, and B. Niknahad. Partition coefficient n-octanol/water of propranolol and atenolol at different temperatures: Experimental and theoretical studies. *Journal of Chemical Thermodynamics*, 54:393–397, 2012.

[43] Ganesh Kamath, Navendu Bhatnagar, Gary A. Baker, Sheila N. Baker, and Jeffrey J. Potoff. Computational prediction of ionic liquid 1-octanol/water partition coefficients. *Physical Chemistry Chemical Physics*, 14(13):4339–4342, 2012.

[44] Navendu Bhatnagar, Ganesh Kamath, Issac Chelst, and Jeffrey J. Potoff. Direct calculation of 1-octanol-water partition coefficients from adaptive biasing force molecular dynamics simulations. *Journal of Chemical Physics*, 137(1), 2012.

[45] Jong Sung Park, Nam Guk Her, and Yeomin Yoon. Sonochemical degradation of chlorinated phenolic compounds in water: Effects of physicochemical properties of the compounds on degradation. *Water, Air, and Soil Pollution*, 215(1-4):585–593, 2011.

[46] Paul C. Stein, Massimiliano Di Cagno, and Annette Bauer-Brandl. A novel method for the investigation of liquid/liquid distribution coefficients and interface permeabilities applied to the water-octanol-drug system. *Pharmaceutical Research*, 28(9):2140–2146, 2011.

[47] Francesca Palombo, Thierry Tassaing, Marco Paolantoni, Paola Sassi, and Assunta Morresi. Elucidating the association of water in wet 1-octanol from normal to high temperature by near- and mid-infrared spectroscopy. *Journal of Physical Chemistry B*, 114(28):9085–9093, 2010.

[48] Collin D. Wick, Bin Chen, and Kalliat T. Valsaraj. Computational investigation of the influence of surfactants on the air-water interfacial behavior of polycylic aromatic hydrocarbons. *Journal of Physical Chemistry C*, 114(34):14520–14527, 2010.

[49] Ioannis G. Economou, Nuno M. Garrido, and Zoi A. Makrodimitri. Prediction of microscopic structure and physical properties of complex fluid mixtures based on molecular simulation. *Fluid Phase Equilibria*, 296(2):125–132, 2010.

[50] Nasser Goudarzi and Mohammad Goodarzi. Application of successive projections algorithm (SPA) as a variable selection in a QSPR study to predict the octanol/water partition coefficients (Kow) of some halogenated organic compounds. *Analytical Methods*, 2(6):758–764, 2010.

[51] Adel Noubigh, Arbi Mgaidi, and Manef Abderrabba. Temperature effect on the distribution of some phenolic compounds: An experimental measurement of 1-octanol/water partition coefficients. *Journal of Chemical and Engineering Data*, 55(1):488–491, 2010.

[52] Nuno M Garrido, António J Queimada, Miguel Jorge, Eugénia A Macedo, and Ioannis G Economou. 1-octanol/water partition coefficients of n-alkanes from molecular simulations of absolute solvation free energies. *Journal of Chemical Theory and Computation*, 5(9):2436–2446, 2009.

[53] Jyotsnendu Giri, Mamadou S. Diallo, William A. Goddard, Nathan F. Dalleska, Xiangdong Fang, and Yongchun Tang. Partitioning of poly(amidoamine) dendrimers between n-octanol and water. *Environmental Science and Technology*, 43(13):5123–5129, 2009.

[54] Patrick S. Redmill, Shannon L. Capps, Peter T. Cummings, and Clare McCabe. A molecular dynamics study of the Gibbs free energy of solvation of fullerene particles in octanol and water. *Carbon*, 47(12):2865–2874, 2009.

[55] Nadia Carmosini and Linda S. Lee. Partitioning of fluorotelomer alcohols to octanol and different sources of dissolved organic carbon. *Environmental Science and Technology*, 42(17):6559–6565, 2008.

[56] Ben Nanzai, Kenji Okitsu, Norimichi Takenaka, Hiroshi Bandow, and Yasuaki Maeda. Sonochemical degradation of various monocyclic aromatic compounds: Relation between hydrophobicities of organic compounds and the decomposition rates. *Ultrasonics Sonochemistry*, 15(4):478–483, 2008.

[57] Justin L. MacCallum, W. F Drew Bennett, and D. Peter Tieleman. Partitioning of amino acid side chains into lipid bilayers: Results from computer simulations and comparison to experiment. *Journal of General Physiology*, 129(5):371–377, 2007.

[58] Stephen H. White. Membrane protein insertion: The biology-physics nexus. *Journal of General Physiology*, 129(5):363–369, 2007.

[59] Tiejun Cheng, Yuan Zhao, Xun Li, Fu Lin, Yong Xu, Xinglong Zhang, Yan Li, Renxiao Wang, and Luhua Lai. Computation of octanol-water partition coefficients by guiding an additive model with knowledge. *Journal of Chemical Information and Modeling*, 47(6):2140–2148, 2007.

[60] Christina Mintz, Michael Clark, William E. Acree, and Michael H. Abraham. Enthalpy of Solvation Correlations for Gaseous Solutes Dissolved in Water and in 1-Octanol Based on the Abraham Model. *Journal of Chemical Information and Modeling*, 47(1):115–121, 2007.

[61] Marie Held and Dan V. Nicolau. Estimation of atomic hydrophobicities using molecular dynamics simulation of peptides. *BioMEMS and Nanotechnology III*, 6799(December 2007):679916, 2007.

[62] Uezu Kazuya and Yoshizuka Kazuharu. Computational Chemistry in Solvent Extraction. *Solvent Extraction Research and Development*, 14:1–15, 2007.

[63] Siewert J. Marrink, H. Jelger Risselada, Serge Yefimov, D. Peter Tieleman, and Alex H. De Vries. The MARTINI force field: Coarse grained model for biomolecular simulations. *Journal of Physical Chemistry B*, 111(27):7812–7824, 2007.

[64] Jahanbakhsh Ghasemi and Saadi Saaidpour. Quantitative structure-property relationship study of n-octanol-water partition coefficients of some of diverse drugs using multiple linear regression. *Analytica Chimica Acta*, 604(2):99–106, 2007.

[65] Fábio M. Engelmann, Silvia V O Rocha, Henrique E. Toma, Koiti Araki, and Maurício S. Baptista. Determination of n-octanol/water partition and membrane binding of cationic porphyrins. *International Journal of Pharmaceutics*, 329(1-2):12–18, 2007.

[66] Pál Jedlovszky and Lívia B. Pártay. Adsorption of octyl cyanide at the free water surface as studied by Monte Carlo simulation. *Journal of Physical Chemistry B*, 111(21):5885–5895, 2007.

[67] Alexandre Chapeaux, Luke D. Simoni, Mark A. Stadtherr, and Joan F. Brennecke. Liquid phase behavior of ionic liquids with water and 1-octanol and modeling of 1-octanol/water partition coefficients. *Journal of Chemical and Engineering Data*, 52(6):2462–2467, 2007.

[68] Raeanne L Napoleon and Preston B Moore. Structural characterization of interfacial n-octanol and 3-octanol using molecular dynamic simulations. *The Journal of Physical Chemistry B*, 110(8):3666–3673, 2006.

[69] Bin Chen and J Ilja Siepmann. Microscopic structure and solvation in dry and wet octanol. *The Journal of Physical Chemistry B*, 110(8):3555–3563, 2006.

[70] Joseph W. Lambert and Amadeu K. Sum. Molecular dynamics study of the properties of capsaicin in an 1-octanol/water system. *Journal of Physical Chemistry B*, 110(5):2351–2357, 2006.

[71] Yuan H. Zhao and Michael H. Abraham. Octanol/water partition of ionic species, including 544 cations. *Journal of Organic Chemistry*, 70(7):2633–2640, 2005.

[72] César Augusto Fernandes De Oliveira, Cristiano Ruch Werneck Guimarães, Heloisa De Mello, Aurea Echevarria, and Ricardo Bicca De Alencastro. A molecular dynamics study on liquid 1-octanol. Part 3. Evaluating octanol/ water partition coefficients of novel thrombin inhibitors via free-energy perturbations. *International Journal of Quantum Chemistry*, 102(5 SPEC. ISS.):542–553, 2005.

[73] Marco Paolantoni, Paola Sassi, Assunta Morresi, and Rosario Sergio Cataliotti. Infrared study of 1-octanol liquid structure. *Chemical Physics*, 310(1-3):169–178, 2005.

[74] Thomas W Jabusch and Deborah L Swackhamer. Partitioning of polychlorinated biphenyls in octanol/water, triolein/water, and membrane/water systems. *Chemosphere*, 60(9):1270–1278, 2005.

[75] William M. Meylan and Philip H. Howard. Estimating octanol-air partition coefficients with octanol-water partition coefficients and Henry's law constants. *Chemosphere*, 61(5):640–644, 2005.

[76] Paola Sassi, Marco Paolantoni, Rosario Sergio Cataliotti, Francesca Palombo, and Assunta Morresi. Water/alcohol mixtures: A spectroscopic study of the water-saturated 1-octanol solution. *The Journal of Physical Chemistry B*, 108(50):19557–19565, 2004.

[77] William H Steel, Carmen L Beildeck, and Robert A Walker. Solvent polarity across strongly associating interfaces. *The Journal of Physical Chemistry B*, 108(41):16107–16116, 2004.

[78] Swen Rabe, Ulrich Krings, and Ralf G. Berger. Dynamic flavour release from Miglyol/water emulsions: Modelling and validation. *Food Chemistry*, 84(1):117–125, 2004.

[79] Ilan Benjamin. Polarity of the water/octanol interface. *Chemical Physics Letters*, 393(4-6):453–456, 2004.

[80] Pál Jedlovszky, Imre Varga, and Tibor Gilányi. Adsorption of 1-octanol at the free water surface as studied by Monte Carlo simulation. *Journal of Chemical Physics*, 120(24):11839–11851, 2004.

[81] X. Zhang, W. H. Steel, and R. A. Walker. Probing solvent polarity across strongly associating solid/liquid interfaces using molecular rulers. *Journal of Physical Chemistry B*, 107(16):3829–3836, 2003.

[82] Michael H. Abraham, Andreas M. Zissimos, Jonathan G. Huddleston, Heather D. Willauer, Robin D. Rogers, and William E. Acree. Some novel liquid partitioning systems: Water-ionic liquids and aqueous biphasic systems. *Industrial and Engineering Chemistry Research*, 42(3):413–418, 2003.

[83] William H Steel and Robert A Walker. Measuring dipolar width across liquid–liquid interfaces with 'molecular rulers'. *Nature*, 424(6946):296, 2003.

[84] Marcela P. Aliste, Justin L. MacCallum, and D. Peter Tieleman. Molecular dynamics simulations of pentapeptides at interfaces: Salt bridge and cation-π interactions. *Biochemistry*, 42(30):8976–8987, 2003.

[85] D. Bas, D. Dorison-Duval, S. Moreau, P. Bruneau, and C. Chipot. Rational determination of transfer free energies of small drugs across the water-oil interface. *Journal of Medicinal Chemistry*, 45(1):151–159, 2002.

[86] César Augusto Fernandes de Oliveira, Cristiano Ruch Werneck Guimarães, and Ricardo Bicca de Alencastro. Molecular dynamics study on liquid 1-octanol. *International Journal of Quantum Chemistry*, 80(45):999–1006, 2000.

[87] David W. Roberts. Application of octanol/water partition coefficients in surfactant science: A quantitative structure-property relationship for micellization of anionic surfactants. *Langmuir*, 18(2):345–352, 2002.

[88] N. M. Kovalchuk, V. I. Kovalchuk, and D. Vollhardt. Auto-oscillations of surface tension: Experiments with octanol and hexanol and numerical simulation of the system dynamics. *Colloids and Surfaces A: Physicochemical and Engineering Aspects*, 198-200:223–230, 2002.

[89] Mario Grassi, Nicoletta Coceani, and Lorenzo Magarotto. Modelling partitioning of sparingly soluble drugs in a two-phase liquid system. *International Journal of Pharmaceutics*, 239(1-2):157–169, 2002.

[90] S. W. Peretti, C. J. Tompkins, J. L. Goodall, and A. S. Michaels. Extraction of 4-nitrophenol from 1-octanol into aqueous solution in a hollow fiber liquid contactor. *Journal of Membrane Science*, 195(2):193–202, 2001.

[91] Justin L MacCallum and D Peter Tieleman. Structures of neat and hydrated 1-octanol from computer simulations. *Journal of the American Chemical Society*, 124(50):15085–15093, 2002.

[92] Igor V. Tetko, Vsevolod Yu Tanchuk, and Alessandro E.P. Villa. Prediction of n-Octanol/Water Partition Coefficients from PHYSPROP Database Using Artificial Neural Networks and E-State Indices. *Journal of Chemical Information and Computer Sciences*, 41(3-6):1407–1421, 2001.

[93] David Michael and Ilan Benjamin. Molecular dynamics computer simulations of solvation dynamics at liquid/liquid interfaces. *Journal of Chemical Physics*, 114(6):2817–2824, 2001.

[94] H. Mulder, A. M. Breure, and W. H. Rulkens. Prediction of complete bioremediation periods for PAH soil pollutants in different physical states by mechanistic models. *Chemosphere*, 43(8):1085–1094, 2001.

[95] Carles Curutchet, Modesto Orozco, and F. Javier Luque. Solvation in octanol: Parametrization of the continuum MST model. *Journal of Computational Chemistry*, 22(11):1180–1193, 2001.

[96] Peter I. Nagy and Krisztina Takács-Novák. Theoretical and experimental study on ion-pair formation and partitioning of organic salts in octanol/water and dichloromethane/water systems. *Journal of the American Chemical Society*, 122(28):6583–6593, 2000.

[97] Bin Chen and J. Ilja Siepmann. Partitioning of alkane and alcohol solutes between water and (dry or wet) 1-octanol. *Journal of the American Chemical Society*, 122(27):6464–6467, 2000.

[98] Shiang Tai Lin and Stanley I. Sandler. Multipole corrections to account for structure and proximity effects in group contribution methods: Octanol-water partition coefficients. *Journal of Physical Chemistry A*, 104(30):7099–7105, 2000.

[99] Luke Chimuka, Lennart Mathiasson, and Jan Åke Jönsson. Role of octanol-water partition coefficients in extraction of ionisable organic compounds in a supported liquid membrane with a stagnant acceptor. *Analytica Chimica Acta*, 416(1):77–86, 2000.

[100] Erin M. Duffy and William L. Jorgensen. Prediction of properties from simulations: Free energies of solvation in hexadecane, octanol, and water. *Journal of the American Chemical Society*, 122(12):2878–2888, 2000.

[101] Andrew Pohorille, Michael A Wilson, and Karl Schweighofer. Electrostatic properties of aqueous interfaces probed by small solutes. *AIP Conference Proceedings*, 492(1):492–509, 1999.

[102] David Michael and Ilan Benjamin. Electronic spectra of dipolar solutes at liquid/liquid interfaces: Effect of interface structure and polarity. *Journal of Chemical Physics*, 107(15):5684–5693, 1997.

[103] Nataša Šegatin and Cveto Klofutar. Thermodynamics of the solubility of water in 1-hexanol, 1-octanol, 1-decanol, and cyclohexanol. *Monatshefte für Chemie/Chemical Monthly*, 135(3):241–248, 2004.
